# Radiolabeled Gold Nanoseeds Decorated with Substance P Peptides: Synthesis, Characterization and In Vitro Evaluation in Glioblastoma Cellular Models

**DOI:** 10.3390/ijms23020617

**Published:** 2022-01-06

**Authors:** Francisco Silva, Alice D’Onofrio, Carolina Mendes, Catarina Pinto, Ana Marques, Maria Paula Cabral Campello, Maria Cristina Oliveira, Paula Raposinho, Ana Belchior, Salvatore Di Maria, Fernanda Marques, Carla Cruz, Josué Carvalho, António Paulo

**Affiliations:** 1 Centro de Ciências e Tecnologias Nucleares, Instituto Superior Técnico, Universidade de Lisboa, Campus Tecnológico e Nuclear, Estrada Nacional 10, Km 139.7, 2695-066 Bobadela LRS, Portugal; alicedonofrio@ctn.tecnico.ulisboa.pt (A.D.); mendes.cic@gmail.com (C.M.); catarina.pinto@tecnico.ulisboa.pt (C.P.); ana.sofia.97.m@gmail.com (A.M.); pcampelo@ctn.tecnico.ulisboa.pt (M.P.C.C.); cristinaoliveira@ctn.tecnico.ulisboa.pt (M.C.O.); paular@ctn.tecnico.ulisboa.pt (P.R.); anabelchior@tecnico.ulisboa.pt (A.B.); salvatore@ctn.tecnico.ulisboa.pt (S.D.M.); fmarujo@ctn.tecnico.ulisboa.pt (F.M.); 2 Departamento de Engenharia e Ciências Nucleares, Instituto Superior Técnico, Universidade de Lisboa, Estrada Nacional 10, Km 139.7, 2695-066 Bobadela LRS, Portugal; 3 CICS-UBI-Centro de Investigação em Ciências da Saúde, Universidade da Beira Interior, Av. Infante D. Henrique, 6200-506 Covilhã, Portugal; carlacruz@fcsaude.ubi.pt (C.C.); josueocarvalho@gmail.com (J.C.)

**Keywords:** gold nanoparticles, substance P, radionuclide therapy, glioblastoma, radiobiological effects, microdosimetry simulations

## Abstract

Despite some progress, the overall survival of patients with glioblastoma (GBM) remains extremely poor. In this context, there is a pressing need to develop innovative therapy strategies for GBM, namely those based on nanomedicine approaches. Towards this goal, we have focused on nanoparticles (AuNP-SP and AuNP-SPTyr8) with a small gold core (ca. 4 nm), carrying DOTA chelators and substance P (SP) peptides. These new SP-containing AuNPs were characterized by a variety of analytical techniques, including TEM and DLS measurements and UV-vis and CD spectroscopy, which proved their high in vitro stability and poor tendency to interact with plasma proteins. Their labeling with diagnostic and therapeutic radionuclides was efficiently performed by DOTA complexation with the trivalent radiometals ^67^Ga and ^177^Lu or by electrophilic radioiodination with ^125^I of the tyrosyl residue in AuNP-SPTyr8. Cellular studies of the resulting radiolabeled AuNPs in NKR1-positive GBM cells (U87, T98G and U373) have shown that the presence of the SP peptides has a crucial and positive impact on their internalization by the tumor cells. Consistently, ^177^Lu-AuNP-SPTyr8 showed more pronounced radiobiological effects in U373 cells when compared with the non-targeted congener ^177^Lu-AuNP-TDOTA, as assessed by cell viability and clonogenic assays and corroborated by Monte Carlo microdosimetry simulations.

## 1. Introduction

Glioblastoma multiforme (GBM), classified as a grade IV brain tumor, represents the most frequent brain tumor, accounting for approximately 12–15% of all intracranial neoplasms. Current therapeutic strategies for GBM rely on open surgery, chemotherapy and radiotherapy. Despite some progress in the past 30 years, the overall survival of patients with glioblastoma remains extremely poor. The average lifespan is approximately 15 months after diagnosis, with most patients experiencing tumor relapse and outgrowth within 7–10 months of initial radiation therapy [1].

In this context, there is a pressing need to develop innovative therapy approaches for GBM, to improve clinical outcomes and reduce the rate of relapse and adverse effects intrinsic to the current treatment options. To tackle this goal, new technologies based on nanometer-sized particles (nanoparticles, NPs) have been studied in the last decade as an attractive alternative to develop innovative tools for the treatment and management of brain cancer [2]. For this purpose, NPs allow exploration of a variety of core structures (e.g., an organic polymer or a metallic material) and different targeting moieties for specific accumulation and retention in the tumors (e.g., an antibody or a small peptide). 

Such research efforts led to the development of NanoTherm, which has been approved recently by the European Medicines Agency (EMA) for thermal therapy of GBM [3]. NanoTherm consists of an aqueous dispersion of iron oxide nanoparticles, which is injected into the tumor and, following the application of an alternating magnetic field, releases heat locally. However, the need of a dedicated magnetic field applicator (not available in most hospitals) may restrain the spread out of GBM treatments based on NanoTherm. Besides iron oxide nanoparticles, AuNPs are another class of inorganic nanoparticles that were also studied to develop new approaches to treat GBM, namely as radiosensitizers or drug-delivery carriers, because of their good biocompatibility [4,5,6,7]. 

In particular, AuNPs are very well suited to deliver imaging or therapeutic radionuclides to tumor tissues, taking advantage of a variety of radiolabeling strategies for a stable incorporation of medically relevant radiohalogens or radiometals into the NPs core and/or their targeting moieties [8,9]. Thus, AuNPs carrying therapeutic radionuclides (e.g., ^103^Pd, ^177^Lu, ^211^At, ^225^Ac) started to be envisaged as nanometric seeds for the local delivery of radiation to solid tumors [10,11,12]. This approach is an attractive alternative to classical millimeter-sized brachytherapy seeds, as it allows the irradiation of the tumor cells with β^−^ or α particles instead of the X-rays emitted by the classical seeds. These ionizing particles have a higher linear energy transfer (LET) than X-rays; therefore, radiolabeled AuNPs might offer a better chance to circumvent radiation resistance processes. Moreover, AuNPs solutions are expected to facilitate intratumoral administration procedures when compared with the insertion of classical brachytherapy devices. Therefore, nanoseed-based delivery of radionuclides to tumors might contribute to a more intimate/molecular brachytherapy approach, while allowing a versatile combination with other imaging/therapeutic modalities.

In the past few years, several authors have studied radiolabeled nanoseeds for GBM treatment, based mainly on localized intratumoral approaches, including AuNPs. Many of these studies have focused on α emitters, such as ^211^At, ^223^Ra or ^225^Ac, as this class of radionuclides can provide efficient therapeutic outcomes due to the inherent high LET of heavy α particles [13,14,15,16,17]. However, the limited availability of α emitters still hinders their clinical translation and widespread use. By contrast, several β^−^ emitters have been in clinical use for several years. In particular, the medium-energy β^−^ emitter ^177^Lu (T_½_ = 6.647 d, mean energy = 133.3 keV) is emerging as the most important theranostic radionuclide in nuclear medicine, in combination with the β^+^ emitter ^68^Ga used for PET imaging [8,18,19].

Despite its advantages, ^177^Lu has only rarely been evaluated in the labelling of nanoseeds for GBM treatment. In the first reported work, ^177^Lu was used to label Gd-modified fullerene nanoparticles and the resulting radiolabeled NPs demonstrated a dose-dependent increase in survival in a human GBM orthotopic tumor-bearing mouse model [20]. These Gd-modified fullerene NPs did not contain any GBM-targeting unit to promote the accumulation and retention in the tumor, exploring, alternatively, the enhanced permeability and retention (EPR) effect, that is involved in the passive targeting of leaky tumor tissues [21]. Later on, Ferro-Flores et al. studied ^177^Lu-labeled AuNPs decorated with a cyclic Arg-Gly-Asp (RGD) peptide, recognizing the α_v_β_3_ integrin receptor, which is not specific for GBM but is involved in tumor angiogenesis [22]. Following the intratumoral administration of the AuNPs in a murine C6-glioma xenograft model, the authors verified highest tumor retention and enhanced reduction in tumor growth for ^177^Lu-AuNP-RGD when compared with the same ^177^Lu-labeled AuNPs without the RGD peptide. These results pointed out that the target-specific approach can lead to improved GBM treatments based on intratumoral application of radiolabeled nanoseeds.

Unlike the α_v_β_3_ integrin receptor, the neurokinin-1 receptor (NKR1) is almost exclusively expressed on the glioma cell surface with a consistent overexpression in all primary malignant gliomas [23]. Hence, the NKR1 has emerged as a good candidate for the development of targeted GBM therapies. The main endogenous ligand of NK1R is substance P that belongs to the family of tachykinin peptide neurotransmitters [24]. Substance P (SP) derivatives were labeled with therapeutic radionuclides, including β^−^ (e.g., ^90^Y and ^177^Lu) and α (e.g., ^213^Bi and ^225^Ac) emitters and using 1,4,7,10-tetraazacyclododecane-1,4,7,10-tetraacetic acid (DOTA) and 1,4,7,10-tetraazacyclododecane-1-glutaric acid-4,7,10-triacetic acid (DOTAGA) bifunctional chelators [25,26,27]. Several of these radiolabeled SP peptides underwent clinical trials that involved intratumoral or intracavitary therapy cycles. Encouraging results were obtained in terms of median survival when compared with other therapeutic modalities. 

For an intratumoral approach, radiolabeled nanoseeds are an attractive alternative to more diffusible molecular systems, such as radiolabeled SP peptides, as they should exhibit highest tumor retention with consequent improvement of therapeutic outcomes. Having this in mind, Bilewicz et al. have designed AuNPs carrying a SP derivative (SP(5–11)) and proceeded with their labeling with the α emitter ^211^At. The resulting ^211^At-labeled AuNPs showed high in vitro radiocytotoxicity in T98G glioblastoma cells, moderately larger than that showed in the same cell line by the radiolabeled AuNPs without the SP peptide [14]. On contrary, to our knowledge, there is no study on ^177^Lu-labeled AuNPs functionalized with SP peptides as radionanoseeds for GBM treatment, despite the favorable nuclear properties of ^177^Lu for radionuclide therapy and its greater availability compared with ^211^At. Aiming to capitalize on these advantages, we decided to study ^177^Lu-labeled AuNPs functionalized simultaneously with DOTA chelators for a stable coordination of the radiometal and with SP derivatives for the recognition of GBM cells. 

For our purposes, we have used AuNP-TDOTA nanoparticles, which we have previously reported on [28]. AuNP-TDOTA are nanoparticles with a small-sized core (ca. 4 nm) containing a thiolated DOTA derivative (TDOTA = 2-[4,7-bis(carboxymethyl)-10-[2-(3-sulfanylpropanoylamino)ethyl]-1,4,7,10-tetrazacyclododec-1-yl]acetic acid), covalently linked to their surface by Au-S bonds, which confers a largely negative zeta potential value to the NPs, thus preventing their aggregation in solution. This is a crucial issue, as AuNPs for biomedical applications need to be stable in physiological environments. If needed, several strategies can be employed to improve the colloidal stability of AuNPs gold nanoparticles, such as their functionalization with different biomacromolecules and polymers or with thiol-containing small peptides [29,30,31]. As we have previously shown for bombesin (BBN) derivatives, AuNP-TDOTA can be readily functionalized with bioactive peptides carrying an N-terminal thioctic acid function for their Au-S chemisorption through the terminal disulfide group [28,32]. In the present work, we have used the same strategy to couple the native SP sequence (SP(1–11)) to AuNP-TDOTA, as well as to attach the congener sequence SPTyr8, where the original phenylalanine at the 8-position was replaced by a tyrosine residue. Previously, it has been shown that the introduction of the tyrosine residue in this position of the amino acid sequence does not compromise the biological activity of the peptide [33,34]. By considering the SPTyr8 sequence, we have thought on the possibility of performing its labeling with radioiodine at the tyrosyl residue. In this way, we intended to have the ability to ascertain if the targeting vector, the SP derivative, will travel together in the biological milieu with the AuNP-TDOTA core carrying a trivalent radiometal, such as ^177^Lu. This important issue has seldom been addressed in the preclinical evaluation of target-specific nanoseeds.

In this paper, we describe the synthesis and characterization of the new nanoparticles AuNP-SP and AuNP-SPTyr8, obtained by functionalization of AuNP-TDOTA with the SP and SPTyr8 peptides, including the study of their interaction with human serum albumin (HSA) and human transferrin (hTf). Their labeling with ^67^Ga is also reported, as well as cellular uptake studies of the resulting radiolabeled AuNPs (^67^Ga-AuNP-SP and ^67^Ga-AuNP-SPTyr8) in human GBM cells (U87 and T98G), which were performed to assess the influence of the replacement of the phenylalanine amino acid by the tyrosine on the biological performance of the AuNPs. It is also described the radiolabeling of AuNP-SPTyr8 with ^125^I and ^177^Lu and cellular uptake studies for the respective ^125^I-AuNP-SPTyr8 and ^177^Lu-AuNP-SPTyr8 in the U373 glioblastoma cell line. To have a first insight on the influence of the target-specific approach in terms of therapeutic efficacy of the designed nanoseeds, we also present the study of the radiobiological effects induced by ^177^Lu-AuNP-SPTyr8 in U373 cells, in comparison with ^177^Lu-AuNP-TDOTA, which does not contain the SP derivative. Finally, we report on microdosimetry simulations, which were performed with the aim of obtaining a better understanding of the experimental radiobiological results and at the assessment of the possible contribution of the AuNPs gold core for radio-enhancement effects. 

## 2. Results

### 2.1. Synthesis and Characterization of AuNPs Carrying SP Peptides

**Scheme 1:**
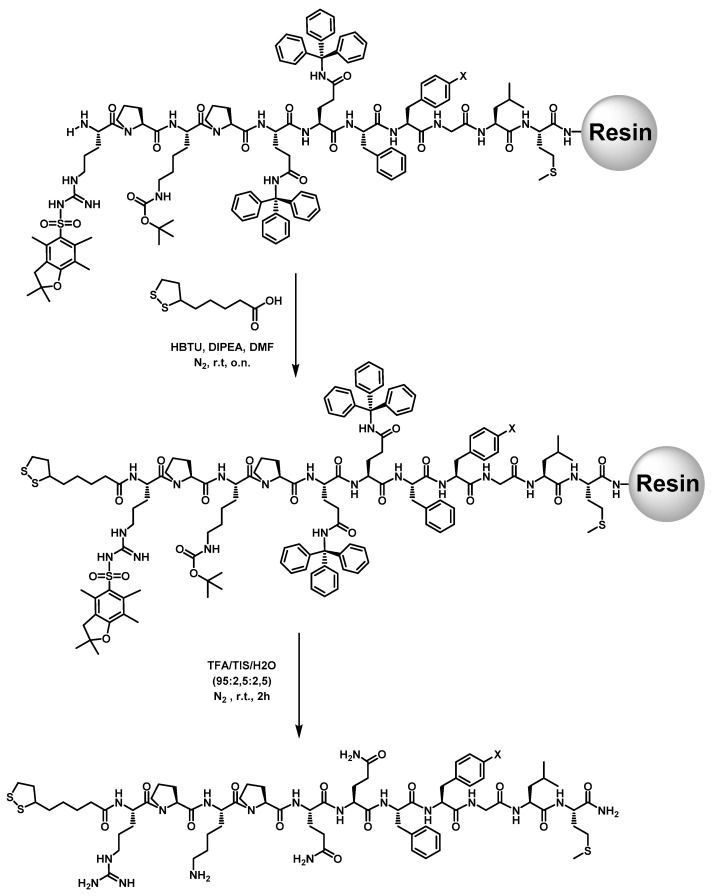
Synthetic route towards the TA-SP (X = H) and TA-SPTyr8 (X = OH) peptides.

The work was initiated with the synthesis of the thioctic acid (TA) derivatives of Substance P peptides, to be used as NK-1 receptor targeting moieties upon attachment to the AuNP-TDOTA nanoconstructs. As depicted in Scheme 1, two different TA-modified peptides were synthesized, containing either the native SP sequence or the modified sequence SPTyr8. In the later sequence, the phenylalanine in the 8-position was replaced by tyrosine to enable a direct ^125^I-labeling of the SP sequence using oxidative methods. The two sequences (SP and SPTyr8) were obtained by solid phase peptide synthesis (SPPS) methodologies in a microwave-assisted automatic synthesizer, as detailed in the experimental section. Then, each peptide sequence, still protected and attached to the resin, was treated with thioctic acid previously activated with HBTU. The resulting TA-terminated SP derivatives (TA-SP and TA-SPTyr8), formed via an amidation reaction, were then deprotected and cleaved from the resin. After HPLC purification, TA-SP and TA-SPTyr8 were characterized by ESI-MS and analytical HPLC. The ESI-MS spectra, acquired in the positive mode, showed prominent protonated ions [M + 2H]^2+^ at m/z 768.9 and 776.4, respectively, with isotope distribution patterns matching well the theoretical ones and in agreement with the assigned structures. The HPLC analysis confirmed that the recovered conjugates had sufficiently high purity to be further used in the functionalization of the AuNP-TDOTA nanoparticles (Figures S1 and S2).

**Figure 1:**
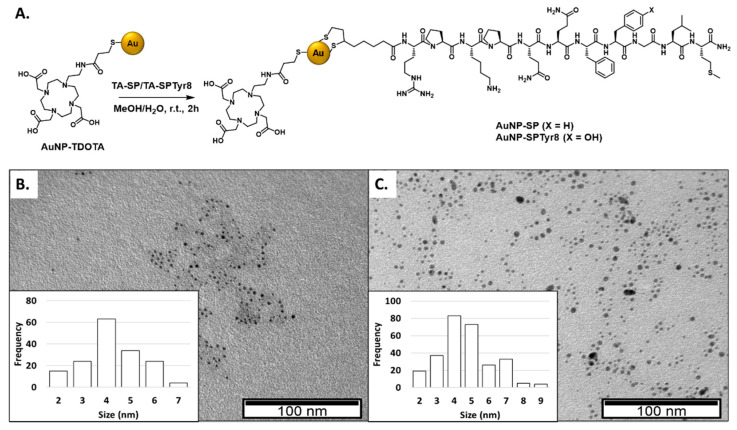
Functionalization of AuNP-TDOTA with the SP peptides to afford AuNP-SP and AuNP-SPTyr8 (**A**). TEM images and corresponding size distribution histograms for AuNP-SP (**B**) and AuNP-SPTyr8 (**C**).

The conjugation of the TA-SP and TA-SPTyr8 peptides to AuNP-TDOTA was performed using experimental conditions similar to those that we have optimized previously, to attach the thioctic derivative of a BBN peptide to the same nanoplatform [28]. Briefly, the conjugation reactions were performed for 2 h at room temperature, using a 2:1 peptide:AuNP mass ratio and taking advantage of the reduction in the disulfide bond of the thioctic ring by the gold atoms resulting into the linkage of the formed dithiolates to the AuNP’s surface (Figure 1). 

The quantities of the TA-SP and TA-SPTyr8 peptides conjugated to AuNP-TDOTA were determined by HPLC analysis of the supernatant of the reaction mixtures, as described in the SM (Figure S3). Under the used experimental conditions, similar conjugation yields of 59% and 62% were achieved for TA-SP and TA-SPTyr8, respectively. The obtained AuNP-SP and AuNP-SPTyr8 contain 0.54 mg and 0.55 mg of peptide per mg of final nanoparticle, respectively. AuNP-SP and AuNP-SPTyr8 were analyzed by the techniques commonly used in the physico-chemical characterization of nanoparticles, which included UV-visible spectroscopy, TEM analysis and zeta potential measurements.

The TEM analysis of the AuNPs carrying the SP derivatives confirmed that the nanoparticles have a small-sized core, between 4 and 5 nm, with a size similar to that exhibited by AuNP-TDOTA (Table 1). The DLS results showed that the hydrodynamic sizes of AuNP-SP and AuNP-SPTyr8 appear in a narrow range, between 93.9 and 96.2 nm, in agreement with their identical payload of peptides with sequences differing only in 1 amino acid. These values are considerably higher than the hydrodynamic size (20.6 nm) of AuNP-TDOTA, reflecting the presence of the peptide layers at the AuNPs surface. This trend might reflect the possible occurrence of polymerization processes during the AuNPs functionalization with the bioactive peptides, as thioctic acid derivatives have the tendency to undergo spontaneous polymerization either in solution or on surfaces [35,36]. Additionally, the zeta potential values of −40.9 and −38.9 mV measured for AuNP-SP and AuNP-SPTyr8, respectively, are less negative than that we have previously reported for AuNP-TDOTA [18]. This difference can be accounted by the presence of basic arginine and lysine amino acids in the peptide sequences that, upon protonation, will contribute to render less negative the overall charge provided by the coating of TDOTA chelators. Nevertheless, the highly negative zeta potentials of AuNP-SP and AuNP-SPTyr8 indicate that these SP functionalized AuNPs are suitably stable and dispersible, as evidenced by the TEM images shown in Figure 1.

### 2.2. Interaction with Plasma Proteins 

In biological media, nanoparticles can interact with a variety of biomolecules, namely with plasma proteins that are prone to form the so-called “protein coronas”. It is well documented that this protein corona might have an important influence on the in vivo behavior of nanoparticles, altering their accumulation and clearance on reticuloendothelial system (RES) organs, such as liver and spleen, and/or their targeting characteristics [37]. This is of particular relevance for nanoparticles administered systemically, but the distribution and retention of intratumorally injected nanoparticles can also be affected by the degree of protein adsorption at their surface [38]. 

Taking into consideration the importance of the interaction with plasma proteins in the biological fate of nanoparticles, we have evaluated the interaction of AuNP-SP and AuNP-SPTyr8 with human serum albumin (HSA) and human transferrin (hTf), which are among the most abundant plasma proteins, together with fibrinogen [39]. For this purpose, two different approaches were used. One was based on the quantification of the amount of each protein attached to the AuNPs surface, upon their exposure to physiological concentrations of the proteins, using circular dichroism (CD) analysis. The other approach aimed to evaluate the influence of the exposure to the proteins on relevant physical properties of the nanoparticles, such as their hydrodynamic size and surface plasmon resonance (SPR). 

CD spectroscopy is a very powerful technique to give information about the secondary structures of proteins, i.e., α-helix, β-sheet and random coil, namely using the far UV region of CD spectra between 250 and 190 nm [40]. CD spectroscopy has been used to assess alterations of the proteins structure upon their interaction with AuNPs and/or to quantify the amount of protein present at the AuNPs surface. This quantification can be performed by measuring the CD spectra of the AuNP–protein conjugates themselves, using appropriate calibration curves. However, this direct quantification requires that the presence of the nanoparticles does not interfere with the CD signal of the protein. This is not always the case and, depending on the AuNP’s concentrations, photons scattering can occur and interfere with the CD signal. The CD signal can also be affected by the strong absorbance of nanoparticles in the UV region, where the most intense protein CD bands usually appear. These difficulties can be circumvented through the quantification of the proteins interacting with the AuNPs in an indirect manner, by separation of the AuNP protein conjugates and measurement of the free protein concentration in the solutions pre-incubated with the AuNPs [41]. 

**Figure 2:**
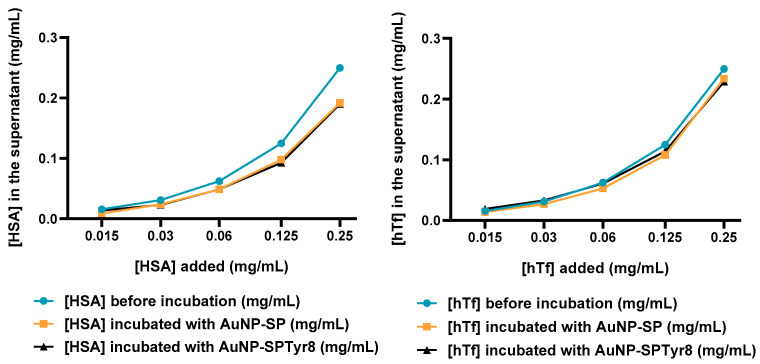
Variation of the concentrations of HSA (**left**) and hTf (**right**) proteins in the supernatant solutions, before and after incubation with AuNP-SP and AuNP-SPTyr8 used at a 0.1 mg/mL concentration, as determined by CD spectroscopy.

In this work, we have used the later approach to quantify the amount of HSA and hTf proteins attached to AuNP-SP and AuNP-SPTyr8, after incubation of solutions of the nanoparticles (0.1 mg/mL) with each protein in the concentration range 0.015–0.25 mg/mL. The NPs were incubated with the protein solutions for 1 h at room temperature and then separated from the mixture by centrifugation. The supernatant was analyzed by CD spectroscopy and the concentration of free protein (HSA or HTf) was determined based on calibration curves obtained for each protein (see Figures S4 and S5). Figure 2 shows the calculated free protein concentration in the supernatant, before and after incubation with the AuNPs, for the tested concentration range. 

As can be verified in Figure 2, the obtained results are relatively identical for AuNP-Tyr and AuNP-SPTyr8, which certainly reflects the similitude of the physico-chemical properties of both nanoparticles, differing uniquely by the replacement of the Phe amino acid by the Tyr amino acid in the SP peptide sequence. However, significant differences were observed in the extent of interaction with each of the evaluated proteins—HSA or hTf. In fact, HSA showed a higher tendency to bind to the AuNPs than hTf in the range of tested concentrations. For the incubating solutions with the highest protein concentration (0.125–0.250 mg/mL), the percentage of attached protein ranged between 21–26% for HSA and between 6–14% for hTf. The reasons for this trend are not clear since the interaction of proteins with AuNPs can be influenced by many factors, such as the size, charge and surface functionalization of the nanoparticles or the physico-chemical characteristics of the proteins (i.e., molecular weight, isoelectric point, structure and folding). However, this trend agrees with the results recently reported by Puntes et al. who verified a higher affinity of HSA for the AuNP surface when compared with plasma globulins [37]. Additionally, other authors reported that pegylated AuNPS can bind to BSA but not to hTf, although the BSA binding can be hampered by the length of the PEG chains [42]. 

**Figure 3:**
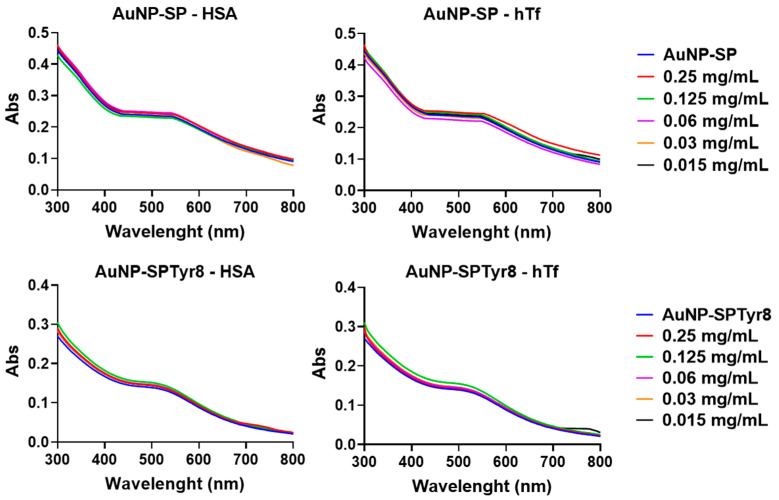
UV-vis spectra of AuNP-SP and AuNP-SPTyr8-([AuNP] = 0.1 mg/mL) exposed to different concentrations of HSA and hTf proteins.

The UV-vis spectra of AuNP-Tyr and AuNP-SPTyr8 in the presence of increasing amounts of HSA and hTf remained almost unaltered, as shown in Figure 3. In particular, there was no increase in the absorbance neither a significant red shift of the plasmon resonance band, as is typically observed when a protein corona is formed around the AuNPs [37,43].

These results pinpoint that the tested proteins have a low tendency to interact with these SP-containing AuNPs. In agreement, the plasmon bands of the AuNPs did not show any broadening, which corroborates that the AuNPs are not undergoing aggregation processes in the presence of HSA and hTf, up to protein concentrations of 0.25 mg/mL. For the same range of concentrations, DLS measurements have shown that the AuNPs retain their monodispersity with a poorly defined variation of their hydrodynamic size with the protein concentration (Figures S6–S9), as reported by other authors for pegylated AuNPs that are not prone to interact with BSA and hTf [42].

### 2.3. Radioiodination (^125^I) and Radiometallation (^67^Ga and ^177^Lu) of the AuNPs

As mentioned in the introductory section, we have proceeded with the ^67^Ga-labeling of the different AuNPs under study, i.e., AuNP-TDOTA, AuNP-SP and AuNP-SPTyr8. ^67^Ga is a trivalent radiometal that is commercially available at economical price and can be stably coordinated by DOTA chelators, such as ^177^Lu. Moreover, ^67^Ga (T_1/2_ = 3.27 d) is a gamma emitter, which is useful for SPECT imaging; therefore, it can form a theranostic pair with ^177^Lu, similarly to the congener ^68^Ga, used for PET imaging [8]. When compared with the short half-life of ^68^Ga (T_1/2_ = 67.8 min), the half-life of ^67^Ga is much better suited to assess the biodistribution of AuNPs, namely, in assessing their tumor retention overtime. As discussed below, these ^67^Ga-labeled AuNPs were used in cellular studies that have led to the selection of AuNP-SPTyr8 for the labeling with ^177^Lu and ^125^I and further biological evaluation.

**Scheme 2:**
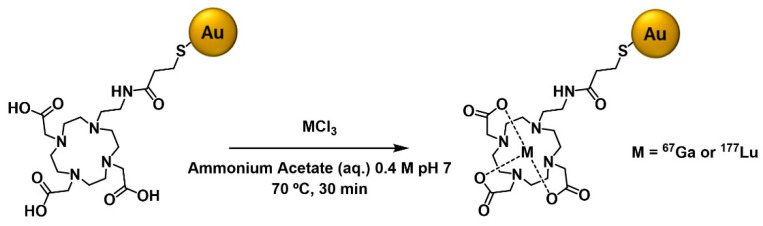
Schematic drawing of the radiolabeling reactions used to obtain radiometallated AuNPs: (i) ^67^Ga-AuNP-TDOTA, ^67^Ga-AuNP-SP and ^67^Ga-AuNP-SPTyr8; (ii) ^177^Lu-AuNP-TDOTA and ^177^Lu-AuNP-SPTyr8 (the gold core generically represents each type of AuNPs, with and without targeting peptide).

The labeling of the AuNPs with the trivalent radiometals was performed as previously reported by us for BBN-containing congeners [28], by reaction with MCl_3_ (^67^Ga or ^177^Lu) under controlled pH (pH ≈ 7) at 70 °C (Scheme 2). After 30 min of reaction, the radiometallated AuNPs were recovered by ultrafiltration and suspended in distilled water. The ^67^Ga- and ^177^Lu-labeled AuNPs were obtained with high radiochemical yield and purity (>95%) (Figures S10 and S11), as assessed by ITLC analysis.

**Scheme 3:**
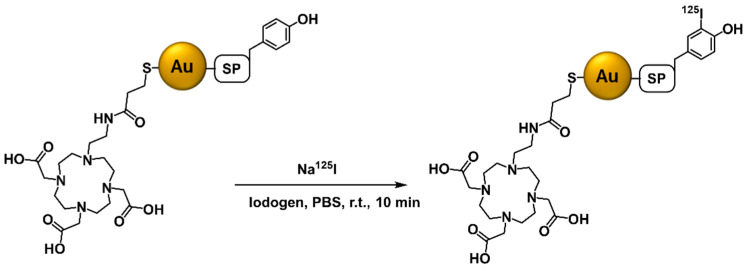
Schematic drawing of the radiosynthesis of ^125^I-AuNP-SPTyr8.

Benefiting from the presence of the tyrosine residue in the modified SP sequence, the AuNP-SPTyr8 nanoparticles were also labeled with ^125^I based on the oxidative radioiodination of the Tyr residue using the Iodogen method (Scheme 3) [44]. The desired radioiodinated nanoparticles (^125^I-AuNP-SPTyr8) were obtained with a high radiochemical yield of 70% under optimized reactions conditions, as described in the experimental section. The radioiodinated AuNPs were successively purified by ultrafiltration and the newly synthesized ^125^I-AuNPs were obtained with excellent radiochemical purity (>95%) (Figure S12), according to their analysis by ITLC chromatography.

**Figure 4:**
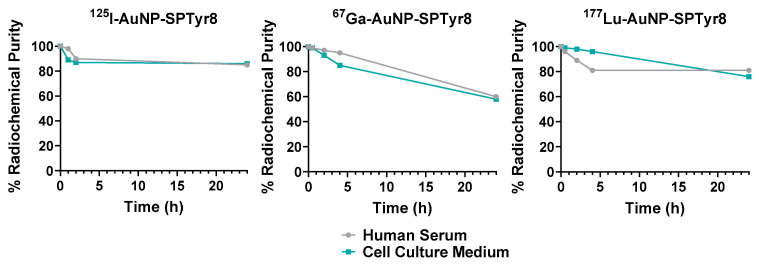
Radiochemical stability of AuNP-SPTyr8 radiolabeled with^125^I, ^67^Ga and ^177^Lu in the presence of human serum and cell culture medium, at 37 °C.

The binding of radionuclides to NPs must be sufficiently stable to prevent their escape from the nanoplatforms in the course of biological studies, either in cellular or animal models, which could lead to misleading results. Thus, it is advisable to perform careful in vitro experiments to assess the stability of radiolabeled NPs, mostly in serum or cell culture medium, prior to their biological evaluation. In this work, the in vitro radiochemical stability of the different radiolabeled AuNPs was evaluated in human serum and culture medium (MEM), prior to their use in further cellular assays. For this study, the AuNPs were incubated with the challenging media at 37 °C, and their radiochemical purity was assessed by chromatographic techniques, as described above. As shown in Figure 4 and Figure S13, all the labeled nanoparticles had a good stability (radiochemical purity >80%) after 4 h of incubation with both media, which corresponds to the longest time point used in the cellular assays described below. This radiochemical stability persisted until 24 h of incubation for ^125^I- and ^177^Lu-AuNP-SPTyr8 but ^67^Ga-AuNP-SPTyr8 presented a larger tendency to release its radiolabel at this late time point.

### 2.4. Cellular Studies

**Figure 5:**
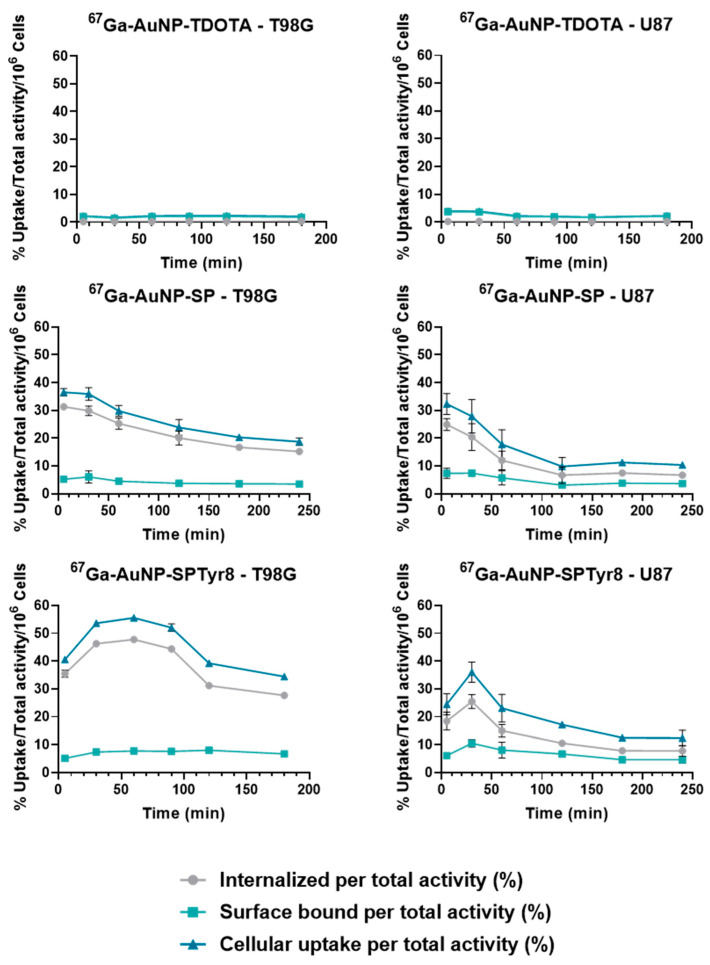
Cellular uptake and internalization of ^67^Ga-AuNP-TDOTA, ^67^Ga-AuNP-SP and ^67^Ga-AuNP-SPTyr8 in T98G and U87 cells at 37 °C and different incubation times, determined by γ-counting measurements. Results are expressed as the percentage of the total (applied) radioactivity (mean ± SD) and normalized per million cells.

The cellular studies were initiated with the ^67^Ga-labeled AuNPs (^67^Ga-AuNP-TDOTA, ^67^Ga-AuNP-SP and ^67^Ga-AuNP-SPTyr8) aiming to address how the presence of the SP peptides at the surface of the nanoparticles influences their internalization by human GBM cells, as well as to assess the effect of replacing the Phe amino acid by the Tyr at the 8-position of the SP sequence. For the different ^67^Ga-labeled AuNPs, the study was initially conducted in the U87 and T98G cell lines (Figure 5), with comprised cellular uptake and internalization assays that were performed by incubation of the cells with the AuNPs at 37 °C for different intervals of time.

The cellular uptake of ^67^Ga-AuNP-TDOTA, without targeting peptide, was found to be negligible in both cell lines. By contrast, ^67^Ga-AuNP-SP and ^67^Ga-AuNP-SPTyr8 showed a fast uptake and internalization in T98G and U87 cells, as can be observed in Figure 5. In the T98G cell line, ^67^Ga-AuNP-SP showed maximum uptake and internalization values of 36.5 ± 1.3% and 31.3 ± 1.1%, respectively, after 5 min of incubation with the cells. In turn, the maximum uptake of ^67^Ga-AuNP-SPTyr8 in T98G was 55.6 ± 0.8% and occurred after 45 min incubation, being observed an internalization of 47.8 ± 0.4% at this time point. A similar behavior was observed for both targeted AuNPs in the U87 cell line, albeit with smaller cellular uptake and internalization when compared with the T98G cell line. In all these assays, the activity retained by the cells decreased with time after reaching plateau values, suggesting the involvement of cellular efflux processes. In general, the ^67^Ga-AuNP-SPTyr8 presented the highest internalization values even for the longest incubation time. For instance, after 180 min of incubation with the T98G cells, ^67^Ga-AuNP-SPTyr8 showed an internalization of 27.7 ± 0.2%, while the internalized fraction of ^67^Ga-AuNP-SP corresponded only to 16.7 ± 0.7%.

All in all, these results suggested that the replacement of the native SP peptide at the AuNPs surface by the SPTyr8 sequence did not compromise their ability to interact with the NK1 receptor and undergo extensive internalization in GBM cells. Then, we proceeded with the studies using the AuNP-SPTyr8 and evaluated their cellular uptake and internalization in the U373 cell line that also overexpresses the NK1R, as assessed by Western blot analysis (Figure S14). For this study, we used the ^125^I-, ^67^Ga- and ^177^Lu-labeled AuNP-SPTyr8 taking advantage of the labeling of the AuNPs core with imaging or therapeutic radiometals and labeling of the SP sequence with radioiodine. 

**Figure 6:**
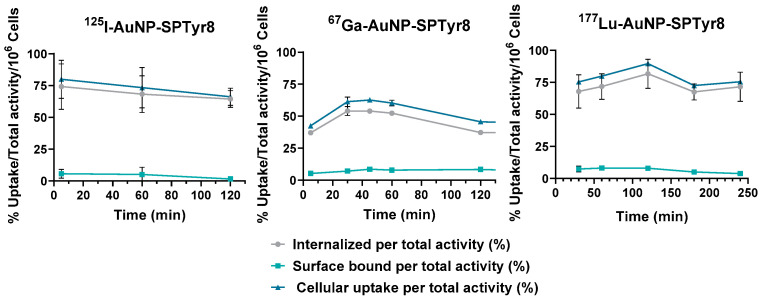
Cellular uptake and internalization of ^125^I-, ^67^Ga- and ^177^Lu-labeled AuNP-SPTyr8 in U373 cells at 37 °C and different incubation times, determined by γ-counting measurements. Results are expressed as the percentage of the total (applied) radioactivity (mean ± SD) and normalized per million of cells.

As can be seen in Figure 6, the ^67^Ga-, ^125^I- and ^177^Lu-labeled AuNP-SPTyr8 have a fast and high cellular uptake and internalization in the U373 cell line. In this cell line, ^67^Ga-AuNP-TDOTA and ^177^Lu-AuNP-TDOTA have a negligible cellular uptake and internalization (Figure S15), which highlights the importance of the presence of the SPTyr8 to promote the internalization of the AuNPs in U373 cells. For AuNP-SPTyr8, labeled with the different radionuclides, the highest internalization rates appear in the range 54.0–67.5%, which are significantly higher than those observed for ^67^Ga-AuNP-SP and ^67^Ga-AuNP-SPTyr8 in T98G and U87 cells. Interestingly, ^125^I-AuNP-SPTyr8 and ^177^Lu-AuNP-SPTyr8 presented very similar cellular uptake and internalization values for the different time points.

**Figure 7:**
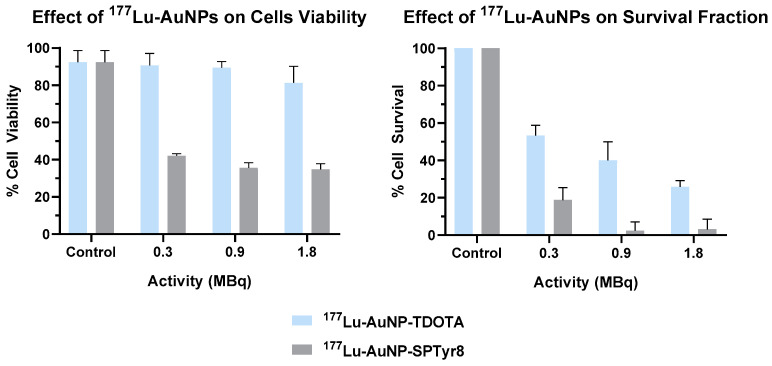
Results of the MTT (**left**) and clonogenic (**right**) assays for U373 cells treated with ^177^Lu-AuNP-TDOTA or ^177^Lu-AuNP-SPTyr8, compared with untreated control cells. Data are expressed as the percentage of cellular viability and percentage of survival fraction, respectively (mean ± SD).

The excellent internalization results obtained for ^177^Lu-AuNP-SPTyr8 in U373 cells prompted us to perform preliminary radiobiological studies for this target-specific ^177^Lu-labeled nanoconstruct, using the same cell line, and in a comparison with the non-targeted congener ^177^Lu-AuNP-TDOTA. These preliminary studies comprised the assessment of the radiocytotoxicity of these ^177^Lu-labeled AuNPs in the U373 cell line using the clonogenic and MTT assays, upon exposure of the cells to increasing activities of the NPs (Figure 7). The clonogenic assay is commonly used to investigate the survival of irradiated cancer cells, either with external radiation or with medical radionuclides, while the MTT assay is often employed to study the cytotoxicity of drugs in human tumoral cells. The clonogenic assay verifies the ability of a single cell to grow into a colony, i.e., to undergo continuous proliferation, and the MTT assay checks the metabolic status of the treated or irradiated cells [45,46].

The U373 cells were incubated with ^177^Lu-AuNP-TDOTA or ^177^Lu-AuNP-SPTyr8 at 37 °C during 4 h, using activities in the range 0.3 to 1.8 MBq. Then, the cell medium containing the radiolabeled AuNPs was replaced by fresh medium and the cell viability was assessed by the MTT assay, after a period of 72 h. As shown in Figure 7, the percentage of cell viability for the U373 cells treated by the non-targeted ^177^Lu-AuNP-TDOTA remained the same (≥90%) as that found for the control cells not treated with the NPs, with exception of the highest activity that led to a very modest decrease in the cell viability. By contrast, the target-specific ^177^Lu-AuNP-SPTyr8 significantly compromised the cell viability of U373 cells in a dose-dependent manner. Their treatment with a 0.3 MBq dose led to a viability reduction of 42.1 ± 1.1% that further decreased to 34.8 ± 3.0% for the highest tested dose (1.8 MBq).

The proliferative status of U373 cells incubated for 4 h with ^177^Lu-AuNP-TDOTA or ^177^Lu-AuNP-SPTyr8, using activities ranging from 0.3 MBq to 1.8 MBq, was evaluated by the clonogenic assay. Thus, after treatment, cells were plated in different dilutions and left to form colonies for 14 days. The results, expressed as survival fraction, are presented in Figure 7. A reduction in the survival fraction was observed even for the lowest activity tested (0.3 MBq) both for the targeted and non-targeted AuNPs, with survival fractions of 53.3 ± 0.05% and 18.8 ± 0.07%, respectively. However, ^177^Lu-AuNP-SPTyr8 caused a much sharper reduction in the survival fraction when compared with ^177^Lu-AuNP-TDOTA, which resulted in a negligible survival fraction (ca. 2%) for the cells treated with the later at a 0.9 MBq dose.

### 2.5. Microdosimetry Studies

Aiming to have an estimation of the absorbed dose for each individual cell in the above described clonogenic and MTT assays, we performed microdosimetry calculations through MC simulations (see Materials and Methods Section), based on the cellular uptake results and the ^177^Lu activities used in the clonogenic and MTT assays. In the case of ^177^Lu-AuNP-TDOTA, where the internalization values were basically close to zero, the culture medium only (CMO) scenario was considered, with 100% of ^177^Lu present in the culture medium and decaying for 4 h (see Table S1). For ^177^Lu-AuNP-SPTyr8 (with an average internalization value of ca. 70%), the following scenarios were taken into account for the clonogenic and MTT assays (see Table 2):

• Scenario 1
Clonogenic assay: 70% ^177^Lu in the nucleus for a decay of 14 days + 4 h, 30% ^177^Lu in the culture medium for 4 h decay;
MTT assay: 70% ^177^Lu in the nucleus for a decay of 72 h + 4 h, 30% ^177^Lu in the culture medium for 4 h decay.
• Scenario 2
Clonogenic assay: 70% ^177^Lu in the cytoplasm for a decay of 14 days + 4 h, 30% ^177^Lu in the culture medium for 4 h decay;
MTT assay: 70% ^177^Lu in the cytoplasm for a decay of 72 h + 4 h, 30% ^177^Lu in the culture medium for 4 h decay.
• Scenario 1/efflux
Clonogenic assay: 70% ^177^Lu in the nucleus for a decay of 4 h, 30% ^177^Lu in the culture media decaying for 4 h (considering efflux, 70% ^177^Lu in the culture media decaying for 14 days);
MTT assay: 70% ^177^Lu in the nucleus for a decay of 4 h, 30% ^177^Lu in the culture media decaying for 4 h (considering efflux, 70% ^177^Lu in the culture media decaying for 72 h).
• Scenario 2/efflux
Clonogenic assay: 70% ^177^Lu in the cytoplasm for a decay of 4 h, 30% ^177^Lu in the culture medium for 4 h decay (considering efflux, 70% ^177^Lu in the culture media decaying for 14 days);
MTT assay: 70% ^177^Lu in the cytoplasm for a decay of 4h, 30% ^177^Lu in the culture medium for 4 h decay (considering efflux, 70% ^177^Lu in the culture media decaying for 72 h).

Looking at the data presented in Table 2, the dosimetric quantities calculated for the clonogenic and MTT assays reflect mainly the duration of each assay (72 h for MTT and 14 days for clonogenic) and the residence time of the radionuclide inside the cells, following the initial incubation period with ^177^Lu-AuNP-SPTyr8. Thus, highest average cell doses were calculated for the clonogenic assay when compared with the MTT assay, either for scenarios without efflux or scenarios where a fast/immediate efflux was considered. For both assays, the scenarios where all the internalized ^177^Lu was located in the nucleus led to greater absorbed dose per cell, in agreement with the MIRD cell S-values data for ^177^Lu [47]. Dosimetric calculations for ^177^Lu-AuNP-SPTyr8 and ^177^Lu-AuNP-TDOTA (Table 2 and Table S1) reasonably support the biological data presented in Figure 7, with more pronounced radiobiological effects for ^177^Lu-AuNP-SPTyr8 being observed, as expected from the calculated cellular absorbed doses. 

**Figure 8:**
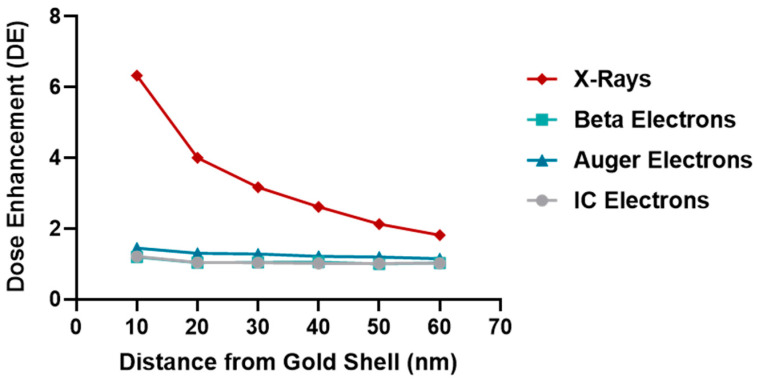
Dose enhancement (DE) factors calculated for the different ^177^Lu radiation emissions, considering the first six water shells with a thickness of 10 nm around the gold shell (see the Materials and Methods Section for geometry setup).

Numerous computational studies have been reported to simulate the radiosensitization properties of gold nanoparticles coupled with external beam radiation [48]. By contrast, the possible radio-enhancement of AuNPs in radionuclide therapy is still poorly studied [49]. In order to assess the possible radio-enhancement effect of coupling AuNPs plus radionuclides, we have performed some MC simulations, according to the geometry setup described in the Materials and Methods Section. Previously, it has been reported that the possible increasing effect on the absorbed dose should be more evident in close proximity to the irradiated AuNP [50]. For this reason, the absorbed dose was calculated in six nanometric shells around the gold shell (see the Materials and Methods Section), with and without gold material, considering the source emitting homogeneously in the cytoplasm. The calculated dose enhancement (DE) factors are presented in Figure 8 for each radiation contribution considered for ^177^Lu emission, as follows: IC electrons, beta, Auger electrons and X-ray. 

For X-ray emission with an average energy of about 2.5 KeV, there is a DE of about 6 in the first 10 nm shell, reaching rapidly the value of about 2 at 60 nm from the gold shell. In this case, the photon energy close to the gold M-shells binding energies can generate a great quantity of low energy Auger electrons that, given their short range (of the order of some nanometer), are able to deposit their energy in close proximity of gold surface. Conversely, Auger, IC and beta radiation seem to have less impact in increasing absorbed dose in presence of gold. As shown in Figure 8, the DE factors for these particles are almost constant (about 1.5) for each water shell considered. Auger and IC electrons have too low energies to cause further electrons cascades in gold material; whereas, the energy of beta radiation (average energy of 140 KeV) generate Auger cascades with less efficiency with respect to L or M shells [51]. The potential benefit of combining high atomic number nanoparticles with labelled radionuclides, such as ^177^Lu, has already been invoked by other authors [49]. Nevertheless, it is important to remark that the physical absorbed dose enhancement does not necessarily imply an enhancement of radiobiological effect (radiosensitization), and it should be carefully verified for each irradiation setup [52].

## 3. Discussion

Our idea was to promote the increased accumulation of gold nanoparticles in human GBM cells by functionalizing AuNP-TDOTA with SP derivatives. We aimed to enhance the cellular uptake of radionuclides carried by the resulting target-specific AuNPs. For this purpose, we have taken advantage of the presence of the TDOTA coating that provides stability to the NPs and warrants a stable radiolabeling with a variety of medical radiometals, while still allowing the functionalization of the NPs surface with a reasonably high payload of the targeting vectors. These favorable features prompted us to proceed with the functionalization of the AuNP-TDOTA nanoplatforms with two different thioctic acid derivatives of SP peptides, containing the native SP sequence and the modified SPTyr8 sequence, based on the reaction of the Au atoms at the NPs surface with the disulfide bridges from the incoming peptides. 

The reaction of AuNP-TDOTA with thioctic acid derivatives of the SP and SPTyr8 peptides was followed by HPLC analysis to ascertain the amount of peptide attached to the NPs surface. This study showed that there are 0.54 and 0.55 mg of each peptide per mg of the recovered AuNP-SP and AuNP-SPTyr8, respectively. Considering a spherical form for the NPs gold core, with an approximate 4 nm diameter, it can be estimated that each individual AuNP contains an average of approximately ten peptide molecules (SP or SPTyr8) at its surface. Although being in the same order of magnitude, this value is inferior to that reported by Bilewicz et al. for the attachment of a HS-PEG-SP(5–11) peptide to 5 nm sized AuNPs of the citrate type (ca. 40 peptide molecules per NP) [14]. This difference might reflect the involvement of two Au atoms in the binding of the thioctic acid derivatives used in this work, while the monothiolated sequence HS-PEG-SP(5–11) binds uniquely to one Au atom [14]. However, the formation of chelated dithiolates in AuNP-SP and AuNP-SPTyr8, instead of monodentate thiolates, has the advantage of providing a more stable binding of the SP derivatives to the AuNPs. In this respect, the Reilly’s group has shown that AuNPs functionalized with DOTA-containing polymers through dithiol or multithiol end groups have greatest stability in vitro and the lowest liver uptake in vivo when compared with the congener AuNPs functionalized using single thiols. This trend was attributed to the greatest inertness of the chelated dithiolates and multithiolates in challenge reactions with endogenous thiolated biomolecules (e.g., dithiothreitol, cysteine or glutathione) [53]. 

The designed AuNP-SP and AuNP-SPTyr8 were thought as nanoseeds for internal radiation therapy of glioblastoma, upon labeling with therapeutic radionuclides (e.g., ^125^I and ^177^Lu) and intratumoral administration, in alternative to classical brachytherapy seeds [54]. The decoration of these NPs with SP peptides for NK1R targeting was expected to promote the internalization in glioblastoma cells to prolong tumor retention and enhance radiobiological effects. SP derivatives are known to have a poor metabolic stability due to their rapid degradation (1 to 2 min) by serum peptidases following their entry into the systemic circulation. However, it has been shown that ^90^Y^_^labeled SP derivatives are stable at the target site for up to 72 h, upon intratumoral administration in GBM tumors [1,55]. Besides the possible role of the targeting vector in the intratumoral retention of AuNP-SP and AuNP-SPTyr8, the interaction with plasma proteins might also influence their retention and diffusion in tumors. Thus, we have studied the in vitro interaction of these AuNPs with HSA and hTf proteins, using several analytical techniques (CD and UV-vis spectroscopy and DLS measurements). Overall, the results of these studies indicated that AuNP-SP and AuNP-SPTyr8 have a low tendency to interact with these plasma proteins. In particular, this trend is well supported by the CD results that pinpointed a low protein coverage of the AuNPs under the tested conditions. For HSA, which showed the best ability to interact with the NPs, a rough estimation indicates that such coverage corresponds approximately to 1 protein molecule per 10 NPs, based on the HSA molecular weight (66,348 Da) and amount of adsorbed protein according to the CD experiments. These findings certainly reflect the presence of a considerable payload of TDOTA chelators and SP peptides at the surface of the AuNPs that minimize their interaction with the proteins and avoid aggregation processes, as verified by CD and DLS measurements. 

The favorable physico-chemical properties and high in vitro stability of the AuNP-SP and AuNP-SPTyr8 nanoparticles prompted us to pursue their labeling with medical radionuclides (^67^Ga, ^177^Lu and ^125^I). The labeling with the trivalent radiometals ^67^Ga and ^177^Lu proceeded well with radiochemical yields >95%, reflecting the suitability of DOTA chelators to coordinate Ga^3+^ and Lu^3+^ ions [56]. For the labeling with ^125^I, we have taken advantage of the presence of a tyrosyl residue in the SPTyr8 sequence that allowed an efficient electrophilic radioiodination of AuNP-SPTyr8 (ca. 70% radiochemical yield), using the Iodogen method as reported by other authors for the ^125^I-labeling of RGD-containing AuNPs [57]. The direct reaction of AuNP-SP or AuNP-SPTyr8 with Na^125^I could be an alternative to label these nanoparticles with ^125^I, as the I^−^ ion can undergo a strong chemisorption on the gold surface with formation of Au-I bonds [58]. However, we have preferred to label the SPTyr8 sequence at the AuNP surface in order to have a better ability to follow the fate of the targeting vector attached to the nanoparticles, while anticipating a stable radiolabeling as typically observed for radioiodinated tyrosine residues. Consistently, ^125^I-AuNP-SPTyr8 and the congener ^177^Lu-AuNP-SPTyr8 have shown excellent radiochemical stability in the presence of human serum and cell culture medium, as the challenge media. Under the same conditions, ^67^Ga-AuNP-SP and ^67^Ga-AuNP-SPTyr8 had a somehow lower radiochemical stability but were still sufficient for their use in subsequent cellular studies. This difference certainly reflects the highest kinetic inertness of Lu–DOTA complexes when compared with the Ga–DOTA congeners, due to the smallest size of Ga^+3^ ions [56].

After confirming the radiochemical stability of the radiolabeled AuNPs, we have pursued with their biological evaluation in GBM cells overexpressing the NKR1. The initial studies were performed with the ^67^Ga-labeled AuNPs and aimed to evaluate how the presence of the two different SP peptides would influence the cellular internalization of the NPs. Thus, the cellular uptake and internalization of ^67^Ga-AuNP-TDOTA, ^67^Ga-AuNP-SP and ^67^Ga-AuNP-SPTyr8 were studied in T98G and U87 cells. The presence of the SP peptides revealed to be crucial to promote the uptake and internalization of the AuNPs by the GBM cells, with maximal internalization values ranging between 20 and 50% of the applied activity per million cells. By contrast, the non-targeted parental nanoplatform (^67^Ga-AuNP-TDOTA) presented a negligible internalization (<1%) in both cell lines. These data indicate that the interaction with the NK1R certainly plays an important role in the uptake and internalization of these SP-containing AuNPs.

Notoriously, the replacement of the phenylalanine amino acid by the tyrosine at the 8-position of the SP sequence did not influence the AuNPs ability to be efficiently internalized by the GBM cells. Therefore, the cellular studies were continued with ^67^Ga-, ^125^I- and ^177^Lu-labeled AuNP-SPTyr8 using the tumorigenic U373 cell line. A fast and high uptake and internalization were observed for ^125^I-AuNP-SPTyr8 and ^177^Lu-AuNP-SPTyr8, with almost equal values for the different time points, reaching plateau values of approximately 80% of the applied activity per million cells. This result seems to corroborate that there is a stable linkage between the targeting vector and the AuNPs surface under biological conditions, as well as a stable binding of the two different radioactive labels (^125^I or ^177^Lu). ^67^Ga-AuNP-SPTyr8 showed a relatively lower cellular internalization with a maximum internalization value close to 60% of the applied activity per million cells, which is probably due to the lowest radiochemical stability of these ^67^Ga-labeled AuNPs in the cell culture media.

The high cellular uptake and internalization of the radiolabeled AuNPs in the U373 cells encouraged the evaluation of the radiotherapeutic effects caused by ^177^Lu-AuNP-SPTyr8 in this cell line. The study was performed in comparison with ^177^Lu-AuNP-TDOTA that was barely taken up or internalized by the U373 cells. In this way, we intended to assess how the degree of internalization would affect the radiobiological effectiveness of the soft β^−^ emitter ^177^Lu. Remarkable differences were observed for the radiobiological effects induced by the non-targeted and targeted nanoparticles, both in the viability and clonogenic assays. ^177^Lu-AuNP-SPTyr8 reduced much more strongly the cell viability and survival in a dose-dependent manner, which highlights the positive influence of the cellular internalization in the radiobiological effects caused by ^177^Lu. For both ^177^Lu-AuNP and ^177^Lu-AuNP-SPTyr8, more notorious radiobiological effects were found in the clonogenic assay than in the MTT assay, as often observed for ionizing radiation [59]. It is important to notice that the “cold” (non-radioactive) AuNP-TDOTA and AuNP-SPTyr8 had no effect in the survival of the U373 cells, even for the maximum gold concentration involved in the assays with the ^177^Lu-labeled congeners (Figure S16). 

Finally, we have performed microdosimetry studies that comprised the assessment of cellular absorbed doses using Monte Carlo simulations, based on the cellular uptake results obtained for ^177^Lu-AuNP-TDOTA and ^177^Lu-AuNP-SPTyr8 and on different scenarios in terms of subcellular localization of the radioisotope and/or its efflux from the target cells. For all the considered scenarios, the calculated doses for ^177^Lu-AuNP-SPTyr8 were considerably higher than those calculated for the non-internalized ^177^Lu-AuNP-TDOTA. For the clonogenic assay, the ratios of calculated absorbed doses for ^177^Lu-AuNP-SPTyr8 vs. ^177^Lu-AuNP-TDOTA appear in the range 30–450, depending on the considered scenarios. The highest ratios correspond to the scenarios where the ^177^Lu is located in the nucleus without efflux. As for the experimental results, the ratios of the survival fractions in the clonogenic assays for ^177^Lu-AuNP-TDOTA vs. ^177^Lu-AuNP-SPTyr8 spanned between 2.2 and 22.5, attesting the highest radiation dose deposited in the cells by the later in agreement with the Monte Carlo simulations. We should notice that the cellular absorbed doses were calculated uniquely based on physical processes of interaction involving the different type of radiation emitted by ^177^Lu (Auger and internal conversion electrons, β^−^ particles, X-ray and γ photons), without considering chemical and biological processes. In this respect, radiosensitization effects of the gold nanoparticles can also play a role, as invoked by other authors for radiolabeled AuNPs [51]. For these radiosensitization effects, the simulations performed in this work showed that the dose enhancement (DE) factors are more relevant in the case of the X-ray photons emitted by ^177^Lu.

In summary, ^177^Lu-AuNP-SPTyr8 has emerged in this study as a very promising radiolabeled nanoseed for the treatment of localized GBM upon intratumoral administration. Thus, it deserves a further preclinical evaluation that should include radiotherapeutic assays in GBM xenografts, which should be performed in comparison with the non-targeted congener ^177^Lu-AuNP. These studies should confirm if the excellent cellular internalization of ^177^Lu-AuNP-SPTyr8 will translate into augmented tumoral retention and improved therapeutic outcomes. Interestingly, the congener ^125^I-AuNP-SPTyr8 is expected to have a similar tumor retention and biodistribution profile, based on the tracer principle. Thus, AuNP-SPTyr8 can be seen as a versatile nanoseed for the delivery of therapeutic radionuclides with different emission properties to GBM tumors, including classical brachytherapy radioisotopes, such as ^125^I. Compared with traditional brachytherapy seeds, AuNP-SPTyr8 is expected to undergo significant local diffusion from the site of injection with a more uniform dose distribution in the tumor. In addition, it allows a profit from all the particles emitted by the radionuclides to obtain enhanced radiobiological effects, due to the intimate molecular contact of the nanoparticles with the tumor tissues. On the contrary, classical seeds fully attenuate short-range particles, such as Auger and internal conversion electrons, capitalizing mainly on penetrating radiation, such as that in X-rays. 

## 4. Materials and Methods

### 4.1. General Procedures

All chemicals and solvents were of reagent grade and were used without further purification, unless stated otherwise, and were commercially acquired from Aldrich Chemical Co. Solvents for high-performance liquid chromatography (HPLC) were HPLC-grade. For the preparation of aqueous solutions and for rinsing of gold nanoparticles, Milli-Q (DI) water (ρ < 18MΩ) was used. The amino acids used in this work were acquired from Novabiochem. The AuNP-TDOTA nanoparticles were synthesized according to previously published methods [28]. ^67^GaCl_3_ was prepared from ^67^Ga-citrate (acquired from Mallinckrodt) following a protocol previously described [60]. Sodium [^125^I] iodide was obtained from Perkin Elmer, USA, as a non-carrier added solution in 0.1 M aqueous NaOH with radionuclidic purity >99% and specific activity of 643.8 GBq/mg. [^177^Lu]Cl_3_ as a carrier-added (specific activity >500 GBq/mg Lu) solution in 0.04 M HCl was kindly provided by the Radioisotope Centre POLATOM, National Centre for Nuclear Research in Otwock, Poland. 

### 4.2. Synthesis of Thioctic Acid (TA)-Terminated SP Derivatives: TA-SP and TA-SPTyr8

The SP (Arg^1^-Pro^2^-Lys^3^-Pro^4^-Gln^5^-Gln^6^-Phe^7^-Phe^8^-Gly^9^-Leu^10^-Met^11^-NH_2_) and SPTyr8 (Arg^1^-Pro^2^-Lys^3^-Pro^4^-Gln^5^-Gln^6^-Phe^7^-Tyr^8^-Gly^9^-Leu^10^-Met^11^-NH_2_) sequences were obtained by solid phase peptide synthesis (SPPS) methodology employing a standard Fmoc strategy in an automated peptide synthesizer (Liberty; CEM), as we have described previously, using microwave irradiation and rink amide resin (MBHA) [28]. Thereafter, for each sequence of interest, 50 mg of resin was swelled with 3 mL of dichloromethane (DCM) for 10 min. The DCM was removed and 2 mL of dimethylformamide (DMF) and 16 µL of N,N-diisopropylethylamine (DIPEA) were added. In a separate flask, 20 mg of thioctic acid was dissolved in 3 mL of DMF and 34 mg of O-(benzotriazol-1-yl)-N,N,N′,N′-tetramethyluronium hexafluorophosphate (HBTU) was added. After 5 min, the mixture was added to the resin solution and the reaction was left overnight, under nitrogen bubbling at room temperature. Then, the supernatant was removed and the cleavage of the peptide from the resin was performed using a 2 × 2 mL solution of TFA/Tris/H_2_O (95:2.5:2.5). The final peptide was precipitated with diethyl ether, centrifuged for 5 min at 3000 rpm, dissolved in a mixture of acetonitrile and H_2_O and purified by HPLC. The HPLC purification was performed in a Perkin-Elmer series 200 equipment, using a Supelco Analytical Discovery Bio C18 column and the following elution conditions: flow rate = 1 mL/min; gradient elution with a mixture of aqueous 0.1% TFA (A) and ACN + 0.1% TFA (B). Method: 0–5 min, 100% A; 5–15 min, 100–0% A; 15–25 min, 0% A; 25–26 min, 0–100% A; 26–30 min, 100% A. The collected fractions were freeze-dried, after ACN evaporation under a N_2_ stream, to afford the final peptides TA-SP and TA-SPTyr8. The recovered peptides were characterized by ESI-MS and by HPLC analysis using the same method as that used in their purification.

TA-SP: η = 11%; ESI(+)-MS m/z calcd for [C_71_H_111_N_18_O_14_S_3_]^+^ = 1535.8; found: m/z [M + H]^+^ = 1535.6; calcd [M + 2H]^2+^ = 768.4; found [M + 2H]^2+^ = 768.9; HPLC (λ 254 nm): Rt = 15.46 min.

TA-SPTyr8: η = 14%; ESI(+)-MS m/z calcd for [C_71_H_112_N_18_O_15_S_3_]^2+^ = 776.4; found [M + 2H]^2+^ = 777.0; HPLC (λ 254 nm): Rt = 16.42 min.

### 4.3. Synthesis of the AuNPs Functionalized with SP Peptides: AuNP-SP and AuNP-SPTyr8

MeOH 1(50 µL) was added to 150 µL of an AuNP-TDOTA solution (5 mg/mL in DI water) and then 300 µL of the desired TA-peptide solution (5 mg/mL, in MeOH) was added. The mixture was stirred at room temperature for 2 h. The solution was centrifuged at 12,000 rpm for 5 min and the pellet was washed with MeOH and H_2_O and lyophilized, to afford the nanoconjugates AuNP-SP and AuNP-SPTyr8. As described below, the characterization of AuNP-SP and AuNP-SPTyr8 involved TEM and DLS analysis and zeta potential measurements. The payload of TA-SP and TA-SPTyr8 peptides was assessed by HPLC analysis, as detailed in the Supplementary Materials.

### 4.4. Electrospray Ionization Mass Spectrometry (ESI-MS)

The mass spectra were obtained in an ESI/QITMS Bruker HCT spectrometer, using electrospray ionization, in positive ion mode. The samples were dissolved in MeOH or CH_3_CN.

### 4.5. Dynamic Light Scattering (DLS) and Zeta Potential Determination

DLS measurements were carried out in a Malvern Zetasizer Nano ZS (Malvern Instruments Ltd., Worcestershire, UK), equipped with a 633 nm He-Ne laser and operating at a 173° angle. The data were collected and analyzed with the Dispersion Technology Software (DTS) version 5.10 from Malvern. Each sample (600 μL) was measured in low volume semi-micro disposable sizing cuvettes (Fisher Scientific, Waltham, MA, USA) with a 10 mm path length. Measurements were made in triplicate at a position of 4.65 mm from the cuvette wall with an automatic attenuator. For each sample, 15 runs of 10 s were performed. For all nanoparticle samples, the autocorrelation function in the “general purpose mode” was used to obtain the size distribution, the Z-average diameter and the polydispersity index (PDI), with a default filter factor of 50% and a default lower threshold of 0.05 and upper threshold of 0.01. Zeta potential measurements were run in triplicate with water as the dispersant and using the Huckel model. For each sample, 20 runs were performed in auto-analysis mode.

### 4.6. Transmission Electron Microscopy (TEM)

TEM images were obtained on a JEOL 1400 transmission electron microscope, JEOL LTD., Tokyo, Japan. The samples were prepared by placing the gold nanoparticle solution (5 μL) on the 300-mesh, carbon-coated copper grid. Then, the excess solution was carefully removed, and the grid was allowed to dry for 5 min. The average size and size distribution of the nanoparticles were determined by processing the TEM images using Adobe Photoshop with Fovea plug-ins.

### 4.7. Interaction with Plasma Proteins

Solutions of human serum albumin (HSA) and human transferrin (hTf) were prepared in concentrations ranging from 0.25 to 0.015 mg/mL after serial dilution in PBS. Each sample was then incubated at RT for 1 h under gentle mixing in the presence of 0.1 mg/mL of AuNPs. Then, the AuNP–protein conjugates were separated from the supernatant by centrifugation at 12,000× *g* for 20 min. Supernatant was analyzed by circular dichroism (CD) and the concentration of free protein was determined from calibration curves established for the range of tested protein concentrations by measuring the ellipticity at 208 nm (see the SM, Figures S4 and S5). CD analysis was performed on a Jasco J-815 spectropolarimeter equipped with a Peltier temperature controller (model CDF-426S/15).

The solutions of AuNP-SP and AuNP-SPTyr8 in the presence of HSA and hTf were analyzed by DLS and UV-vis spectroscopy to assess any aggregation processes. The DLS measurements were performed as described above. The UV-visible absorption spectra were recorded at room temperature using a Cary 60 UV-vis spectrophotometer (Agilent Technologies) in quartz cuvettes with a 10 mm path length.

### 4.8. Radiolabeling of AuNPs with ^67^Ga, ^177^Lu and ^125^I

#### 4.8.1. Radioactive Activity Measurements and Radiochromatography Analysis

The radioactive activities of the radioactive solutions containing ^125^I, ^67^Ga or ^177^Lu were measured in an ionization chamber (Aloka, Curiemeter IG-C3). 

The radiolabeling reactions were monitored by instant thin layer chromatography (ITLC) using glass microfiber chromatography paper impregnated with silica gel (ITLC-SG) (Agilent Technologies). MeOH/6M HCl (95:5) was used as eluent system. The ITLC plates were scanned with a miniGITA TLC scanner (Elysia-Raytest; Straubenhardt, Germany), and the resulting chromatograms were analyzed by GINA-STAR software (Elysia-Raytest) for identification of the different peaks and integrating the area under the curve (AUC) for each peak. The radiochemical purity of the purified radiolabeled AuNPs (R_f_ = 0) was determined by radio-TLC analysis using the same chromatographic system considering that under these conditions the free radioisotopes (^125^I, ^67^Ga or ^177^Lu) show R_f_ = 1.0.

#### 4.8.2. Labeling with ^67^Ga

In a 2 mL eppendorf, 350 µL of 0.4 M ammonium acetate (pH ≈ 7) were mixed with 20 µL of the desired AuNPs (5 mg/mL in DI water) (AuNP-TDOTA, AuNP-SP and AuNP-SPTyr8). Then, 250 µL of ^67^GaCl_3_ in 0.1 M HCl (37–74 MBq) were added. The mixture was heated at 70 °C for 30 min and, after cooling to RT, radio-TLC analysis showed that a radiochemical yield >95 % was obtained. The solution was filtered in a Millipore Amicon Ultra 0.5 mL 10k and the filtered gold nanoconstructs washed with H_2_O (2 × 400 µL) and finally recovered from the filter in 150 μL of H_2_O. Thereafter, radio-TLC analysis showed that ^67^Ga-AuNP-TDOTA, ^67^Ga-AuNP-SP and ^67^Ga-AuNP-SPTyr8 were obtained with a radiochemical purity >95 %.

#### 4.8.3. Labeling with ^177^Lu

In a 2 mL eppendorf, 100 µL of 0.4 M ammonium acetate (pH ≈ 7) were mixed with 5 µL of the desired AuNPs (5 mg/mL in DI water) (AuNP-TDOTA and AuNP-SPTyr8). Following the addition of 3 µL of ^177^LuCl_3_ in 0.04 M HCl (37–74 MBq), the mixture was heated at 70 °C. After cooling to RT, radio-TLC analysis showed that a radiochemical yield >95 % was obtained. The solution was filtered in a Millipore Amicon Ultra 0.5 mL 10k and the filtered gold nanoconstructs washed with H_2_O (2 × 400 µL) and finally recovered from the filter in 150 μL of H_2_O. Thereafter, radio-TLC analysis showed that ^177^Lu-AuNP-TDOTA and ^177^Lu-AuNP-SPTyr8 were obtained with a radiochemical purity >95 %.

#### 4.8.4. Labeling with ^125^I

The procedures were performed in a glove box with negative pressure and suitable for the manipulation of ^125^I. Into a glass vial coated with 100 µg of Iodogen^®^ were added 12 µL of AuNP-SPTyr8 solution (10 mg/mL in DI water) and 128 µL of PBS. Successively, 10 µL of Na^125^I solution (8.7 MBq) were added to obtain a final volume of 150 µL. The reaction was allowed to proceed at room temperature for 10 min. After this reaction time, radio-TLC analysis of the mixture showed a radiolabeling yield of 70%. Thereafter, the ^125^I-AuNP-SPTyr8 were purified by ultrafiltration using a Millipore Amicon Ultra 0.5 mL 10k filter, as described above for the ^67^Ga- and ^177^Lu-labeled AuNPs. Radio-TLC analysis showed that ^125^I-AuNP-SPTyr8 were obtained with a radiochemical purity >95 %.

### 4.9. In Vitro Stability Assays

The stability of the radiolabeled nanoparticles was assessed in human serum and cell culture medium (DMEM with 1% FBS (fetal bovine serum) and 1% antibiotics). To the radiolabeled AuNPs (40 μL solution), 160 μL of the different challenging solutions were added, and the resulting mixtures were incubated at 37 °C for different periods of time (0–24 h). For each time point, the mixtures were analyzed by radio-TLC as described above.

### 4.10. Cell Studies

#### 4.10.1. Cell Culture

T98G, U87 and U373 human glioblastoma cell lines were obtained from ATCC. The cells were cultured in MEM medium (Minimum Essential Medium Eagle, containing Gluta-max) supplemented with 10% FBS and kept at 37 °C in a 5% CO_2_ atmosphere. For the U373 cells, the medium was supplemented with 1% MEM non-essential amino acids and 1% sodium pyruvate. When 70 to 80% cell confluence was achieved, the cells were sub-cultured, adhering to standard procedures, as follows: removal of spent medium; cell wash with PBS; addition of trypsin/EDTA solution for 2–10 min; cell re-suspension in fresh media; cell transference into fresh, warmed, new media; incubation at 37 °C. Antibiotics at 1% (penicillin and streptomycin) were added to the cell medium only for the assays.

#### 4.10.2. Western Blot Analysis

The expression of the NKR-1 receptor in the GBM cell lines was confirmed by Western blot analysis (Figure S14), as described by Guerreiro et al. [61]. Briefly, cells were lysed in ice-cold Cell Lytic reagent containing a cocktail of protease inhibitors and protein concentration was quantified using the DC protein assay (Biorad). A measure of 40 micrograms of protein extract were resolved by SDS-PAGE in a 10% acrylamide gel, transferred to a nitrocellulose membrane and the membrane was probed with an anti-NK1R antibody (D-11, Santa Cruz) followed by a HRP-conjugated secondary antibody (Biorad). Western blot signal was detected by enhanced chemiluminescence, using West Pico ECL Western Blotting Substrate (Ther-mo).

#### 4.10.3. Cellular Uptake

The cellular uptake assays of the different synthesized AuNPs were performed in T98G, U87 and U373 cells seeded at a density of 0.2 × 10^6^ million per well in 24-well culture plates and allowed to attach overnight. The cells were incubated at 37 °C for a period from 5 min to 4 h with approximately 200,000 cpm of the labeled AuNPs under study, in 0.5 mL of culture medium (DMEM with Glutamax; 1%FBS and 1% antibiotics). At the desired incubation time, the cells were washed with cold medium to terminate the incubation, washed with 500 µL of glycine buffer (50 mM glycine; HCl/100 mM NaCl, pH 2.8) for 4 min at room temperature; the procedure was repeated a second time and the fraction corresponding to the cell–surface bound radioactivity was collected. The pH was neutralized with 250 µL PBS and the cells were incubated with 500 µL 1M NaOH to promote cell lysis; the fraction corresponding to the internalized radioactivity was collected. The radioactivity associated with each fraction and that in the cells was measured in a gamma counter (LB 2111, Berthold). Assays for each time point were performed in quadruplicate and data are presented as average ± SEM of typically three independent experiments.

#### 4.10.4. Cell Viability Assay (MTT Assay)

The cytotoxicity of ^177^Lu-AuNP-TDOTA or ^177^Lu-AuNP-SPtyr8 was assessed by evaluating their effects on the proliferation of U373 glioblastoma cells using the [1-(4,5-dimethylthiazol-2-yl)-2,5-diphenyl tetrazolium] (MTT) assay. Cells were seeded in 96-well culture plates at a density of 0.2 × 10^5^ cells/well and left to adhere overnight at 37 °C. Cells were then incubated with the desired ^177^Lu-labeled AuNPs during 4 h at 37 °C, in the range of activities 0.3–1.8 MBq. Then, the medium containing the NPs was replaced by fresh culture medium and the cells maintained at 37 °C for 72 h. After this time, the cells were washed with PBS and then incubated with MTT (200 μL of 0.5 mg/mL solution in cell medium without phenol red) for 3 h at 37 °C. The MTT solution was then removed, and the formed insoluble blue formazan crystals were dissolved in DMSO. The absorbance of this colored solution was measured at 570 nm in a plate spectrophotometer (Power Wave Xs; Bio-Tek). Each test was performed with at least 4 replicates. Control experiments were performed without addition of ^177^Lu-labeled NPs, as well as for the corresponding non-radioactive AuNP-TDOTA and AuNP-SPtyr8. The results were expressed as the percentage of the surviving cells in relation to the control, which was incubated without any compound. 

#### 4.10.5. Clonogenic Assay

U373 cells were seeded at a density of 50,000 cells per well in a 24-well plate and allowed to attach overnight. Cells were incubated with several activities (0.3 to 1.8 MBq) of ^177^Lu-AuNP-TDOTA or ^177^Lu-AuNP-SPtyr8 in 0.5 mL of culture medium, for 4 h at 37 °C. Each test was performed with at least 4 replicates. Control experiments were performed without addition of ^177^Lu-labeled NPs, as well as for the corresponding non-radioactive AuNP-TDOTA and AuNP-SPtyr8 used at the highest concentration involved in the assay with the radioactive counterparts. Immediately after incubation, cells were seeded out in appropriate dilution to form colonies, in 2 weeks, with at least 50 cells. Colonies were fixed with methanol: glacial acetic acid (3:1) and stained with Giemsa (4%).

The plating efficiency (PE), the ratio of the number of colonies to the number of cells seeded, and the survival fraction (SF), the number of colonies that arise after treatment of cells, expressed in terms of PE, were obtained following the methodology described in literature [62], as follows:

(1) $$\mathrm{PE}=\frac{\mathrm{number}\;\mathrm{of}\;\mathrm{colonies}\;\mathrm{formed}\;}{\mathrm{number}\;\mathrm{of}\;\mathrm{cells}\;\mathrm{seeded}}\times 100\%$$

(2) $$\mathrm{SF}=\frac{\mathrm{number}\;\mathrm{of}\;\mathrm{colonies}\;\mathrm{formed}\;\mathrm{after}\;\mathrm{treatment}}{\mathrm{number}\;\mathrm{of}\;\mathrm{cells}\;\mathrm{seeded}\times \mathrm{PE}}$$

### 4.11. Cellular Absorbed Dose Assessment

**Figure 9:**
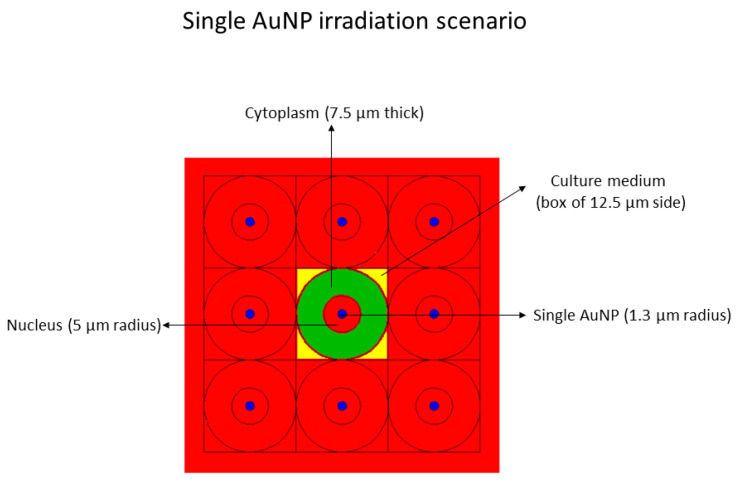
Geometry setup used in the case that a single AuNP is inside the nucleus, and radionuclide emitting homogeneously in the nucleus.

**Figure 10:**
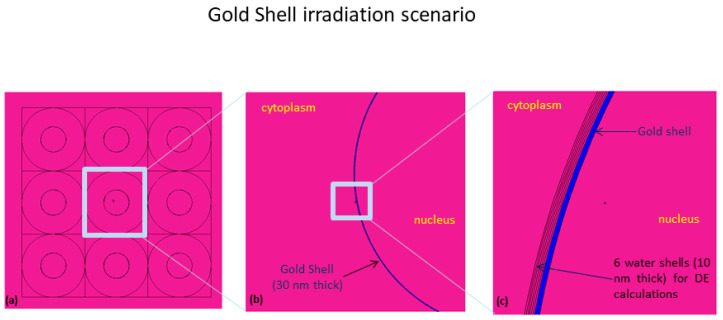
Geometry setup used in the case that the gold shell is around the nucleus, and radionuclide emitting homogeneously in the cytoplasm. (**a**) Entire cell irradiation setup when the emitting source is in the cytoplasm; (**b**) zoom on the gold shell of 30 nm thick around the nucleus; (**c**) zoom near the gold shell to show the nanometric shells used to calculate the dose enhancement (DE) factor.

The absorbed dose assessment in the cell was performed through the state-of-the-art Monte Carlo (MC) code MCNP6.1 [63], largely used in cell dose studies present in the literature [50,52,64]. Since the information about AuNP distribution inside the cell was not available, 2 irradiation scenarios were considered, as follows:

1. Single AuNP at the center in the nucleus, with a radius of 1.3 µm, mimicking 2.83E8 AuNPs, each of 4 nm diameter, with a homogenous radionuclide emitting in the nucleus (see Figure 9).

2. Shell of gold, 30 nm thick, around the nucleus, mimicking 2.83E8 AuNPs, each of 4 nm diameter, with a homogeneous radionuclide distribution emitting in the cytoplasm (see Figure 10).

In both of the above-described scenarios, the culture medium was also considered as an emitting source, according to the different internalization values obtained in the cellular studies. As an example, Figure S17 shows the source emitting in the nucleus and in the culture medium around the cell. The choice of the above irradiation scenarios (single AuNP and gold shell) is largely supported by many studies present in literature [52,65] The single AuNP model mimics a cluster of AuNPs in the nucleus; whereas, the gold shell around the nucleus mimics a preferential AuNPs distribution among the nucleus and cytoplasm, as reported elsewhere [65].

The MC simulation model includes a matrix of nine cells, where the central cell is the emitting one and was modeled as composed by the cytoplasm and nucleus. The nucleus radius is 5 µm, while the entire cell diameter is 25 µm. This model is the same of the cell MIRD one, with the difference that also gold material was included into the cell. The similarity with the MIRD model permits the performance of some comparison among the MIRD results, and consequently can indicate the degree of reasonability of the absorbed dose values obtained in this study. 

The ICRP-107 Auger, internal conversion (IC), β, X-ray and γ emission spectra data were used for this work [66]. Specifically, for IC, X-ray and γ emissions, only the average energy was considered, whereas for β and Auger emissions the entire spectra were considered.

Knowing the initial activity, *A*_0_, used for radiocytotoxicity assays, the time-integrated activity, $\tilde{A}$, for a given Δ*t* was obtained through the following equation:

(3) $$\tilde{A}=1.44T_{p}A_{0}\left(1-\exp\left(-0.693\Delta t/T_{p}\right)\right)$$

where *T_p_* is the physical half-life of ^177^Lu (6.647 days). However, in order to consider the exact activity value in the nucleus or in the cytoplasm, the internalization data were used (see Section 4.10.3). The activity per cell was then obtained by considering 200 µL of medium and a cell diameter of 25 µm.

Finally, some simulations were performed in order to test the physical dose enhancement (DE) capability when ^177^Lu radionuclide is coupled with gold material. The DE factor was calculated in six water shells around the gold shell as showed in Figure 10c. The DE is defined as follows:

(4) $$\mathrm{DE}=\frac{\mathrm{absorbed}\;\mathrm{dose}\;\mathrm{in}\;\mathrm{the}\;i-\mathrm{shell}\;\left(\mathrm{with}\;\mathrm{Gold}\right)}{\mathrm{absorbed}\;\mathrm{dose}\;\mathrm{in}\;\mathrm{the}\;i-\mathrm{shell}\;\left(\mathrm{without}\;\mathrm{Gold}\right)}$$

and was calculated for β, Auger, IC and X-ray contributions emitting in the cytoplasm.

## Figures and Tables

**Table 1:** Physico-chemical characteristics * of AuNP-TDOTA, AuNP-SP and AuNP-SPTyr8 nanoparticles.

AuNP	TEM (nm)	Hydrodynamic Size (PDI) (nm)	Zeta Potential (ζ) (mV)	UV-Vis (nm)
AuNP-TDOTA [28]	4.29 ± 1.60	20.6 (0.3)	−62.6 ± 18.6	520
AuNP-SP	4.24 ± 1.21	93.9 (0.8))	−40.9 ± 10.8	520
AuNP-SPTyr8	4.68 ± 1.51	96.2 (0.7)	−38.9 ± 14.6	520

* All measurements were performed with nanoparticle solutions prepared in DI water (≈pH 6).

**Table 2:** Cell dose assessment for the different scenarios considered for ^177^Lu-AuNP-SPTyr8 (clonogenic and MTT assays).

Applied Activity (MBq)	Average Cell Dose–Scenario 1 (Gy)	Average Cell Dose–Scenario 2 (Gy)	Average Cell Dose-Scenario 1/Efflux (Gy)	Average Cell Dose-Scenario 2/Efflux (Gy)
0.03	6.40 ^a^ 2.33 ^b^	1.51 ^a^ 0.55 ^b^	0.59 ^a^ 0.31 ^b^	0.48 ^a^ 0.20 ^b^
0.9	19.20 ^a^ 7.00 ^b^	4.52 ^a^ 1.66 ^b^	1.78 ^a^ 0.93 ^b^	1.45 ^a^ 0.60 ^b^
1.8	38.39 ^a^ 14.00 ^b^	9.05 ^a^ 3.31 ^b^	3.56 ^a^ 1.86 ^b^	2.90 ^a^ 1.20 ^b^

^a^ clonogenic assay; ^b^ MTT assay.

## Supplementary Materials

The following are available online at https://www.mdpi.com/article/10.3390/ijms23020617/s1.
