# Supplementary material for: Radiolabeled Gold Nanoseeds Decorated with Substance P Peptides: Synthesis, Characterization and In Vitro Evaluation in Glioblastoma Cellular Models
Source: Int J Mol Sci. 2022 Jan 6;23(2):617. doi: 10.3390/ijms23020617 (PMC8775581; doi:10.3390/ijms23020617)
Supplement: Supplementary file 1 [file ijms-23-00617-s001.zip › ijms-1535554-supplementary.pdf]

Supplementary Material

Radiolabeled Gold Nanoseeds Decorated with Substance P Peptides: Synthesis, Characterization and In Vitro Evaluation in Glioblastoma Cellular Models

Francisco Silva *, Alice D'Onofrio, Carolina Mendes, Catarina Pinto, Ana Marques, Maria Paula Cabral Campello, Maria Cristina Oliveira, Paula Raposinho, Ana Belchior, Salvatore Di Maria, Fernanda Marques, Carla Cruz, Josué Carvalho and António Paulo *

FIGURE S1. 2

FIGURE S2. 3

FIGURE S3. 4

FIGURE S4. 5

FIGURE S5. 5

FIGURE S6. 6

FIGURE S7. 9

FIGURE S8. 12

FIGURE S9. 12

FIGURE S10. 13

FIGURE S11. 13

FIGURE S12. 13

FIGURE S13. 14

FIGURE S14. 14

FIGURE S15. 14

FIGURE S16. 15

FIGURE S17. 15

TABLE S1. 15

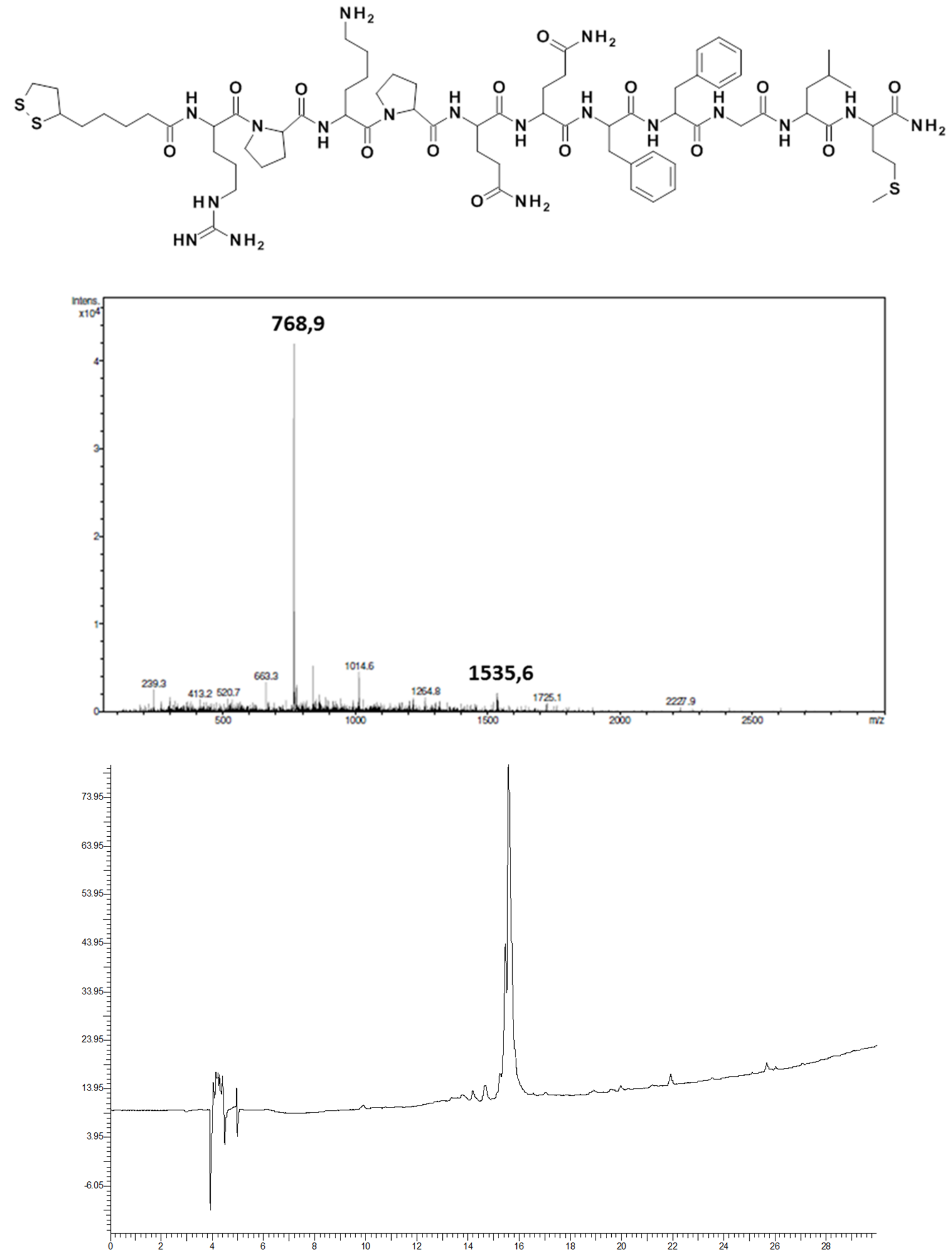

Figure S1. ESI-MS spectrum (positive mode) (m/z: $[C_{71}H_{110}N_{18}O_{14}S_3H]^+$ calc = 1535.8, found = 1535.6; m/z: $[C_{71}H_{110}N_{18}O_{14}S_3H_2]^{2+}$ calc = 768.4; found = 768.9) and HPLC chromatogram (R_t = 15.46 min) for TA-SP.

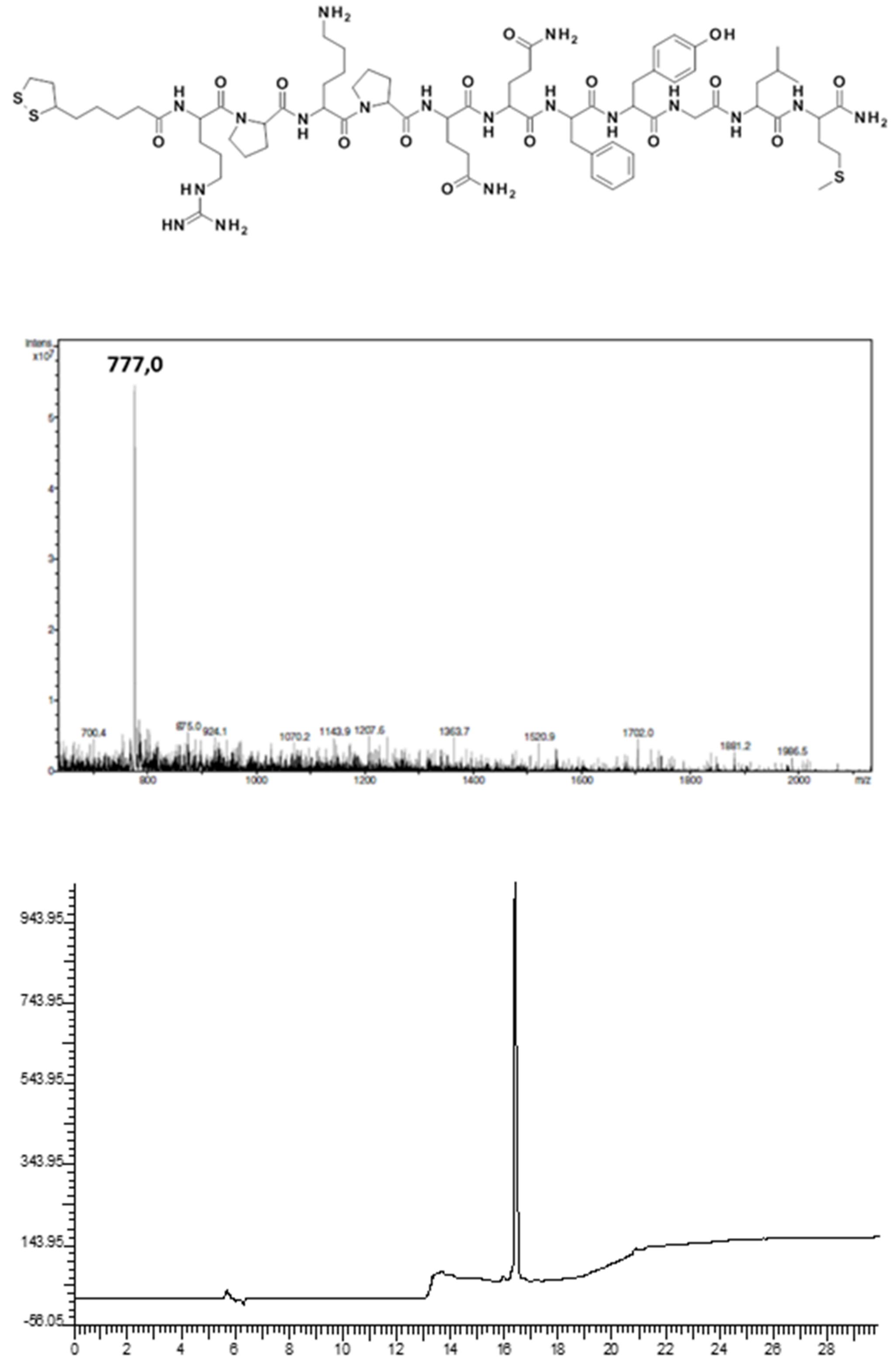

Figure S2. ESI-MS spectrum (positive mode) (m/z: $[C_{71}H_{110}N_{18}O_{15}S_3H_2]^{2+}$ calc = 776.4; found = 777.0) and HPLC chromatogram (R_t = 16.42 min) for TA-[Tyr8]SP.

(a)

Control	C (mg/ml)	Area (µVs)
1	2.5	1405840
2	1.25	738723
3	0.625	394325
4	0.25	143452

Supernatant AuNPs-SP	
Area (µVs)	C (mg/ml)
591337	1.022711

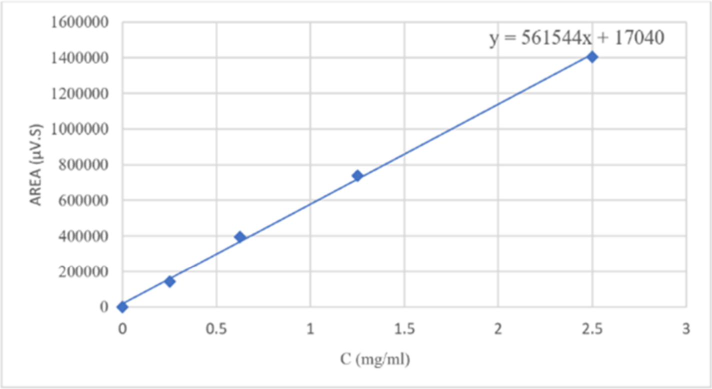

(b)

Control	C (mg/ml)	Area (µVs)
1	0.9	5041335
2	0.45	2566888
3	0.225	1407885
4	0.1125	750550

Supernatant AuNPs-SP	
Area (µVs)	C (mg/ml)
2161630	0.345524

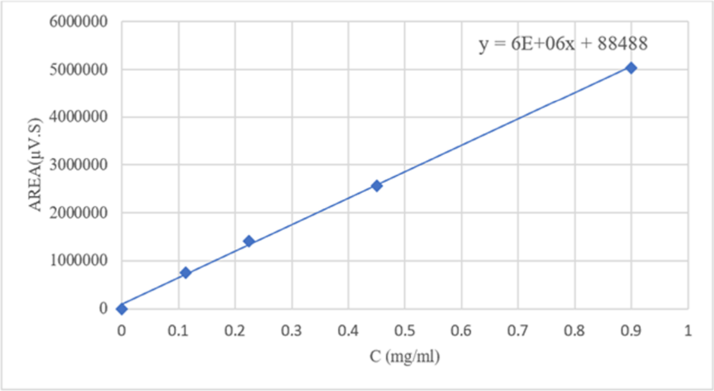

Figure S3. HPLC calibration curve for the determination of (**a**) TA-SP and (**b**) TA-[Tyr8]-SP conjugated to the AuNP-TDOTA nanoparticles (1 mg of AuNP-TDOTA conjugated 1.18 mg of TA-SP and 1.24 mg of TA-[Tyr8]-SP). The quantities of the conjugated peptides were assessed through HPLC control of the supernatant of the reaction mixtures and initial control solutions of the peptides. Peptide control solutions were analyzed by HPLC and the corresponding area under the curve (AUC) was determined. The controls were prepared with progressively halved concentrations.

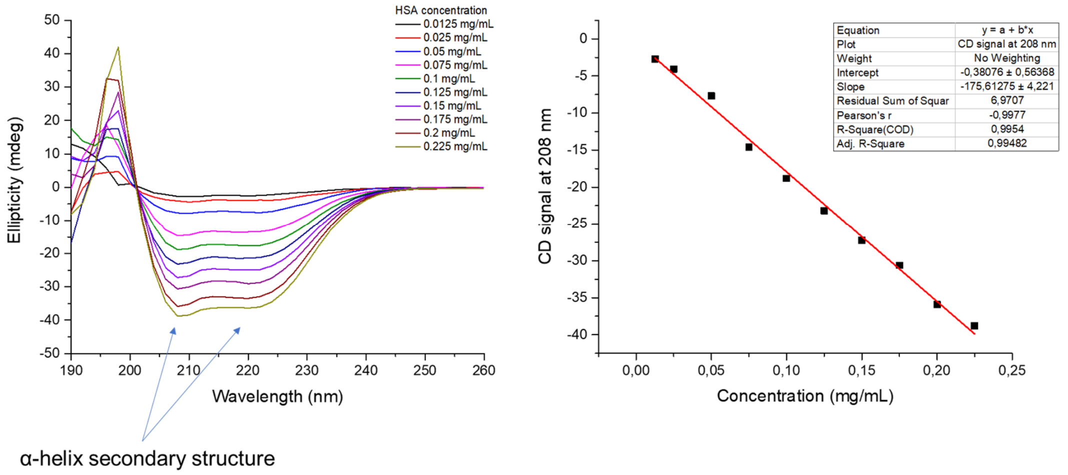

Figure S4. Human Serum Albumin (HSA) calibration curve for the determination of the protein concentration by CD spectroscopy. Protein solutions were prepared in concentrations ranging from 0.25 to 0.0125 mg/mL after serial dilution in PBS from a stock at 10 mg/mL.

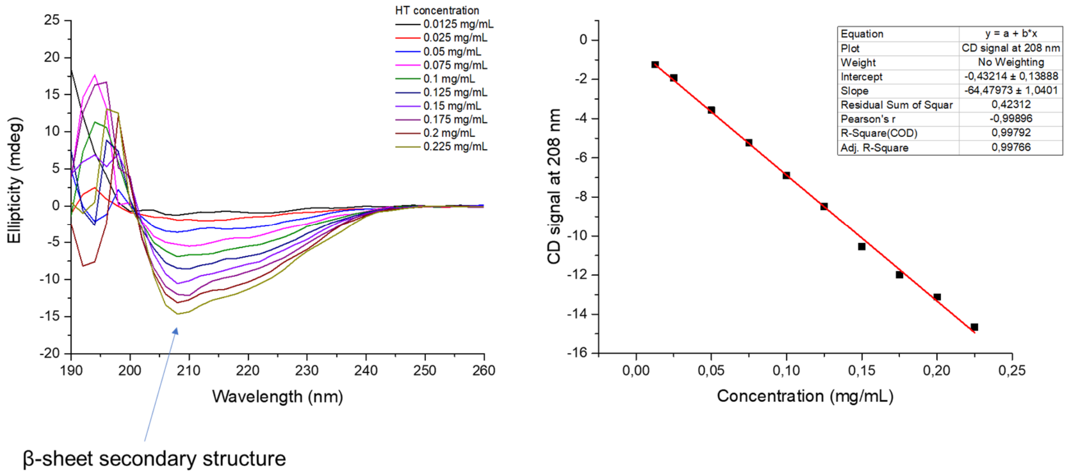

Figure S1. Human Transferrin (hTf) calibration curve for the determination of the protein concentration by CD spectroscopy. Protein solutions were prepared in concentrations ranging from 0.25 to 0.0125 mg/mL after serial dilution in PBS from a stock at 10 mg/mL.

AuNP-SP (no biological substrate)

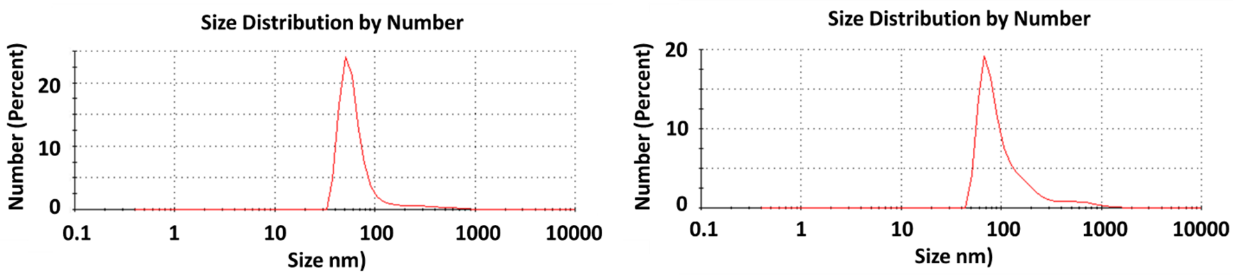

AuNP-SP (0.015 mg/mL HSA

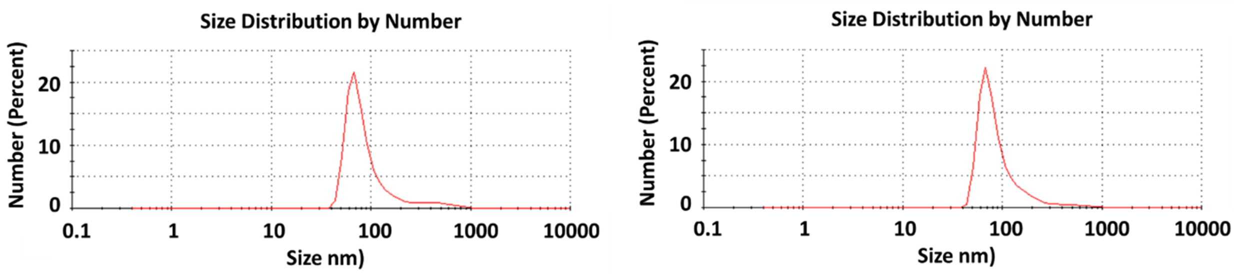

AuNP-SP (0.03 mg/mL HSA)

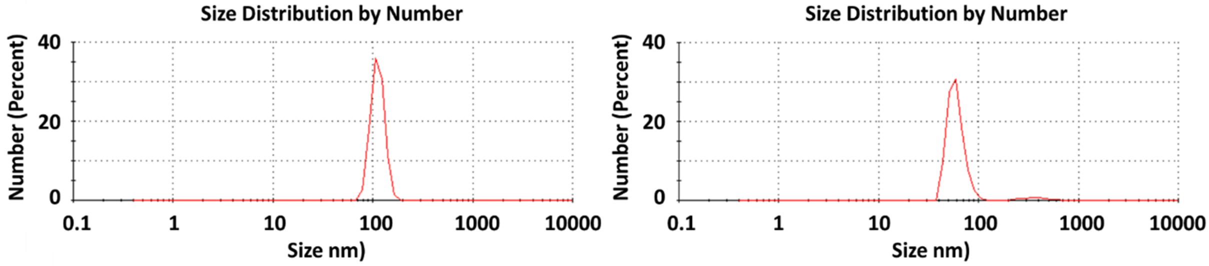

AuNP-SP (0.06 mg/mL HSA)

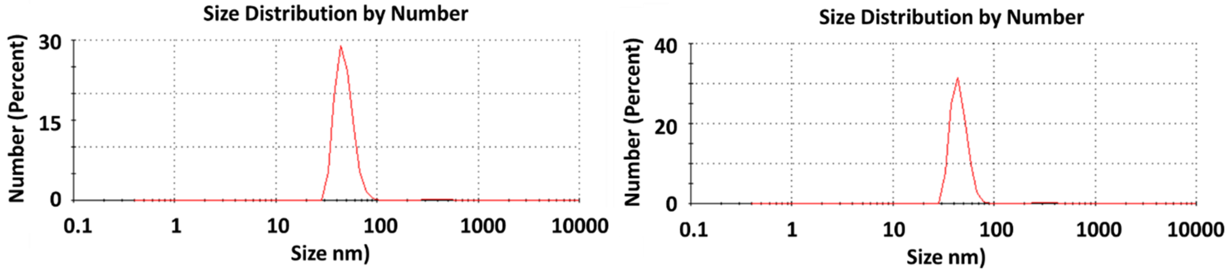

Figure S6. Size distribution by number of particles of AuNP-SP in the presence of HSA and hTf proteins in the concentration range 0.015–0.25 mg/mL. DLS results are given for two independent measurements.

AuNP-SP (0.125 mg/mL HSA)

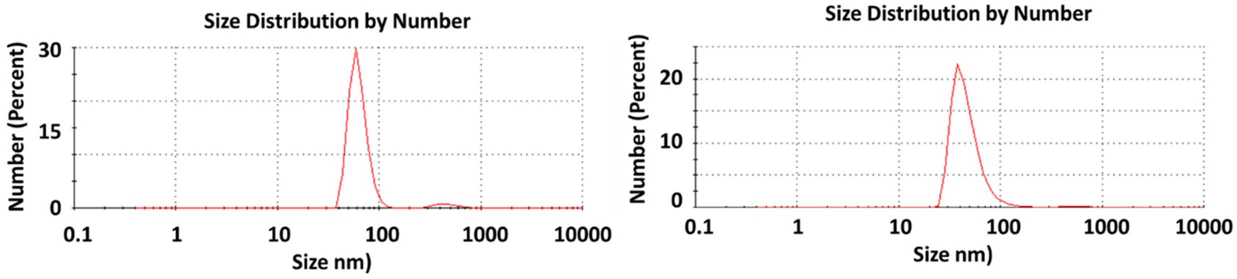

AuNP-SP (0.25 mg/mL HSA)

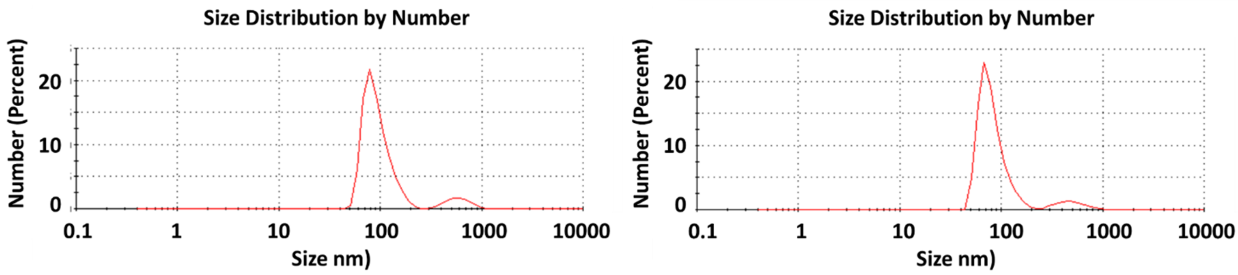

AuNP-SP (0.015 mg/mL hTf)

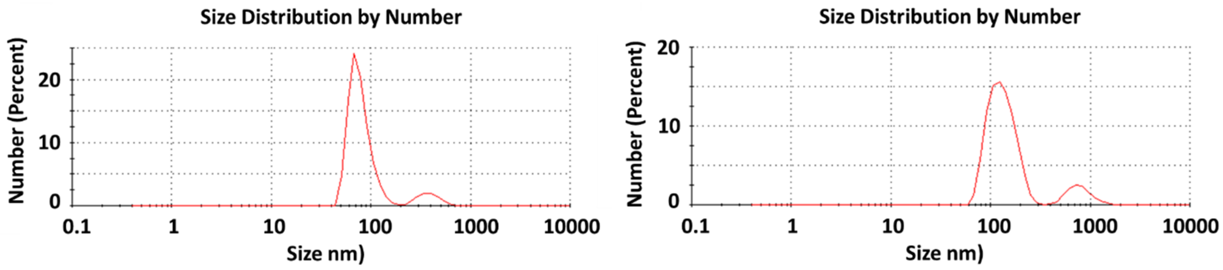

AuNP-SP (0.03 mg/mL hTf)

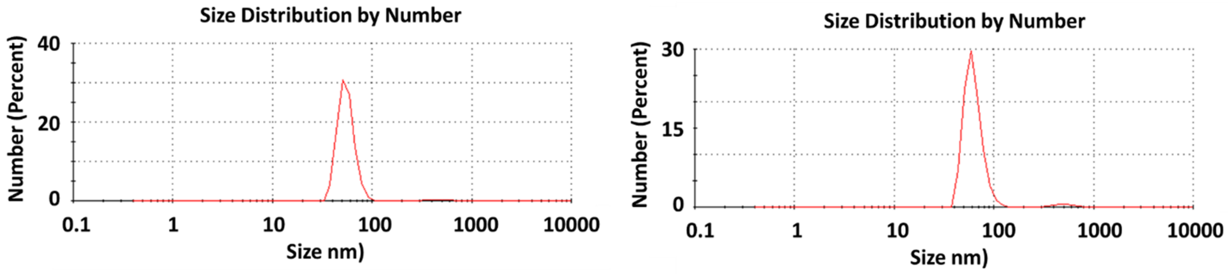

Figure S6. *Cont.* Size distribution by number of particles of AuNP-SP in the presence of HSA and hTf proteins in the concentration range 0.015–0.25 mg/mL. DLS results are given for two independent measurements.

AuNP-SP (0.06 mg/mL hTf)

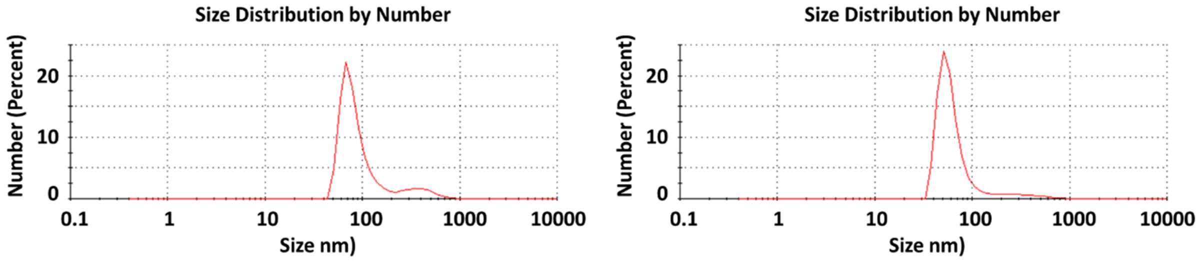

AuNP-SP (0.125 mg/mL hTf)

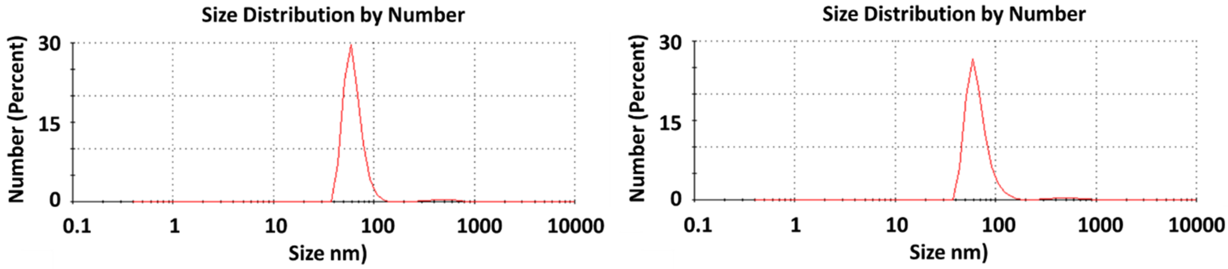

AuNP-SP (0.25 mg/mL hTf):

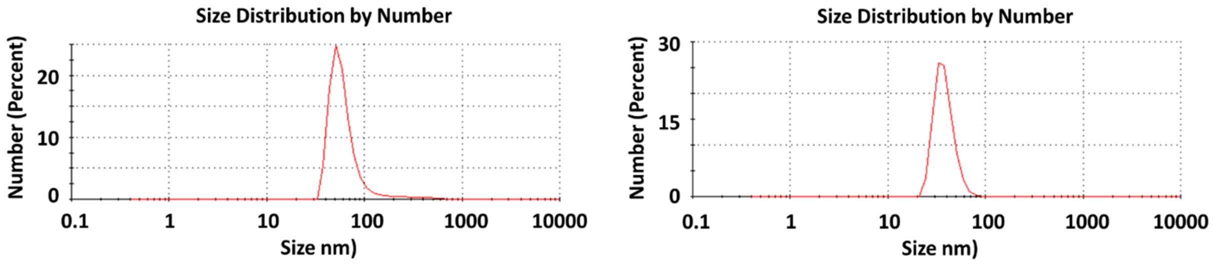

Figure S6. *Cont.* Size distribution by number of particles of AuNP-SP in the presence of HSA and hTf proteins in the concentration range 0.015–0.25 mg/mL. DLS results are given for two independent measurements.

AuNP-SPTyr8 (no biological substrate)

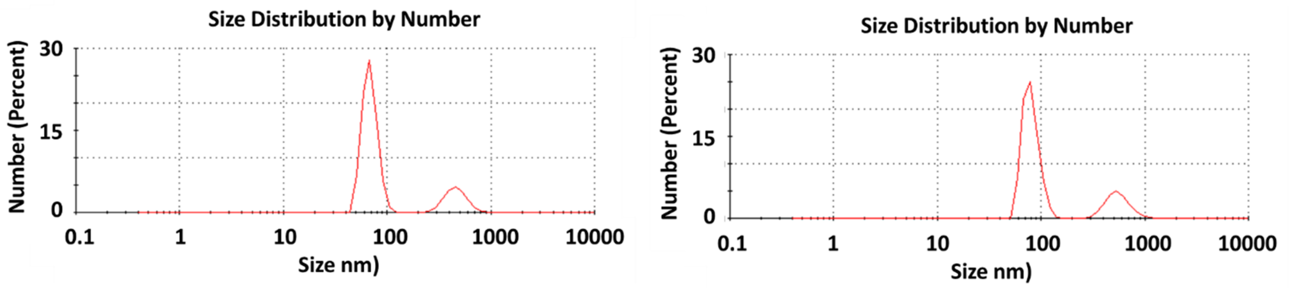

AuNP-SPTyr8 (0.015 mg/mL HSA)

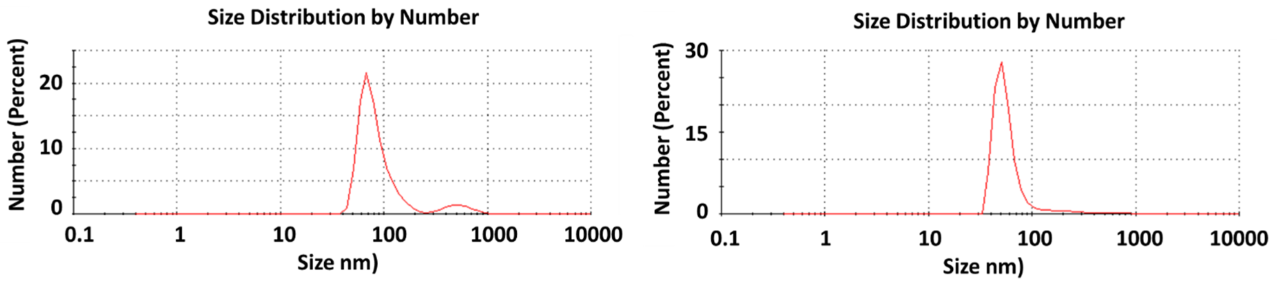

AuNP-SPTyr8 (0.03 mg/mL HSA)

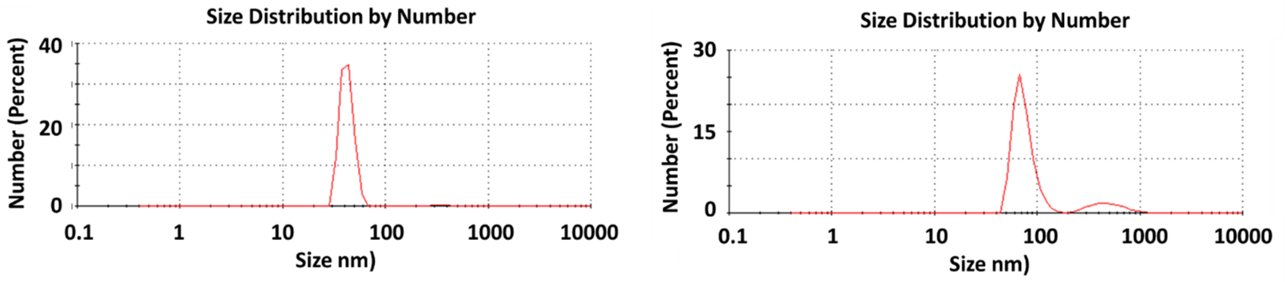

AuNP-SPTyr8 (0.06 mg/mL HSA)

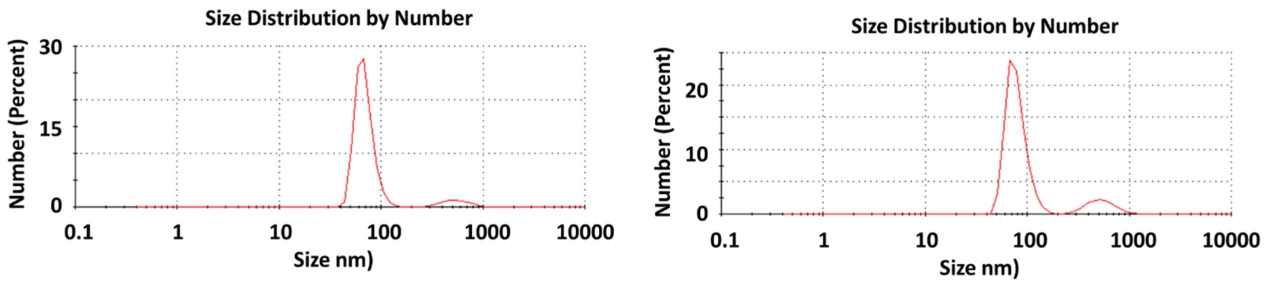

Figure S7. Size distribution by number of particles of AuNP-SPTyr8 in the presence of HSA and hTf proteins in the concentration range 0.015–0.25 mg/mL. DLS results are given for two independent measurements.

AuNP-SPTyr8 (0.125 mg/mL HSA)

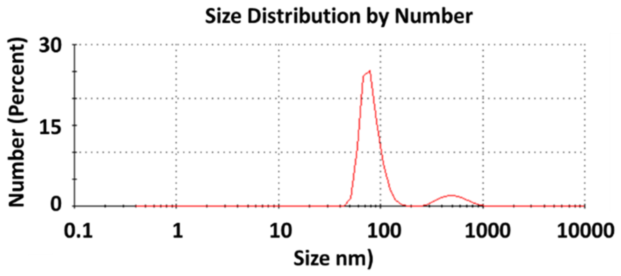

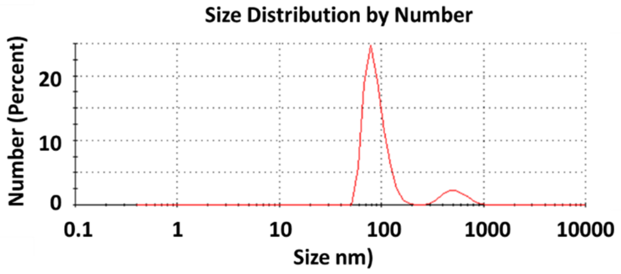

AuNP-SPTyr8 (0.25 mg/mL HSA)

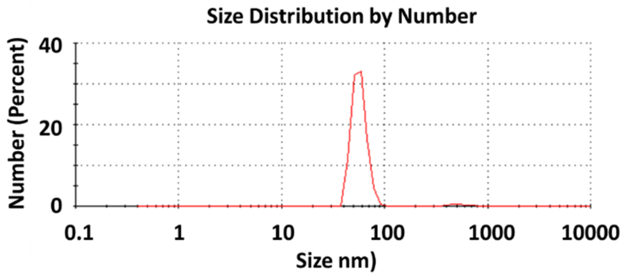

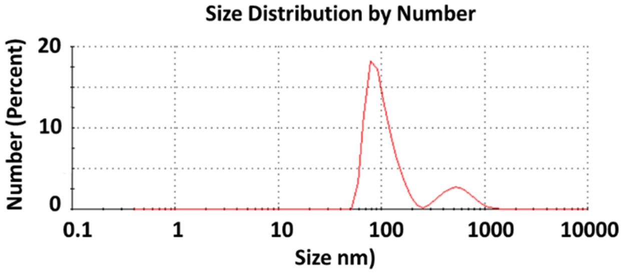

AuNP-SPTyr8 (0.015 mg/mL hTf)

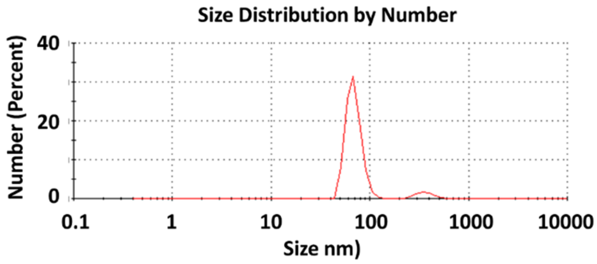

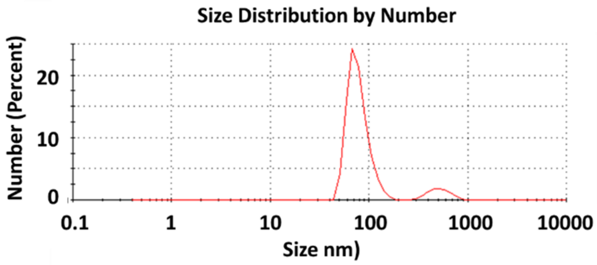

Figure S7. *Cont.* Size distribution by number of particles of AuNP-SPTyr8 in the presence of HSA and hTf proteins in the concentration range 0.015–0.25 mg/mL. DLS results are given for two independent measurements.

AuNP-SPTyr8 (0.03 mg/mL hTf)

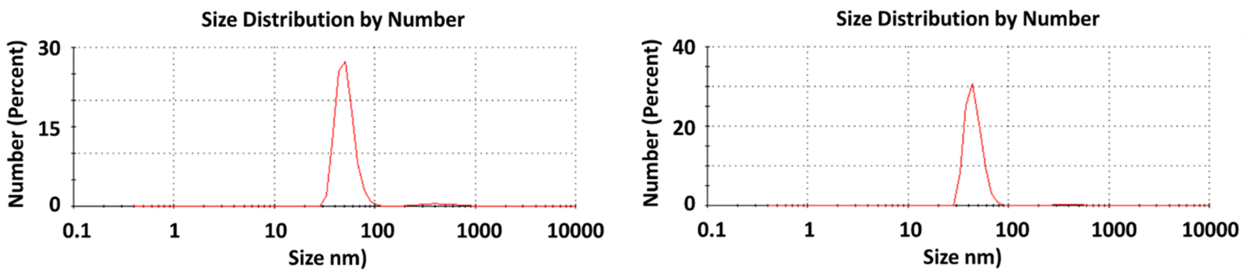

AuNP-SPTyr8 (0.06 mg/mL hTf)

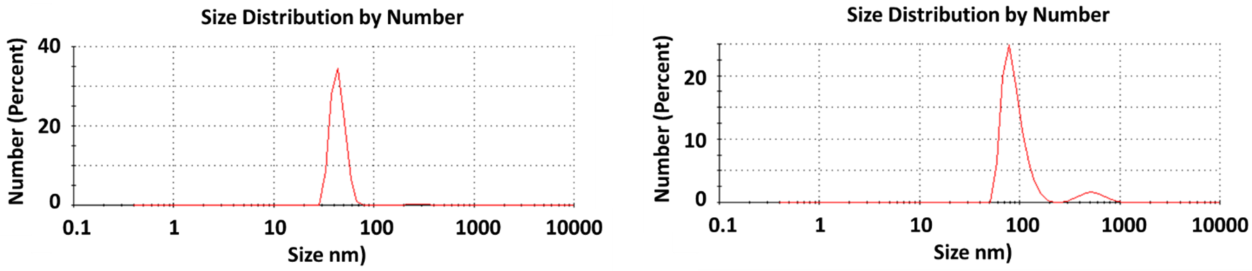

AuNP-SPTyr8 (0.125 mg/mL hTf)

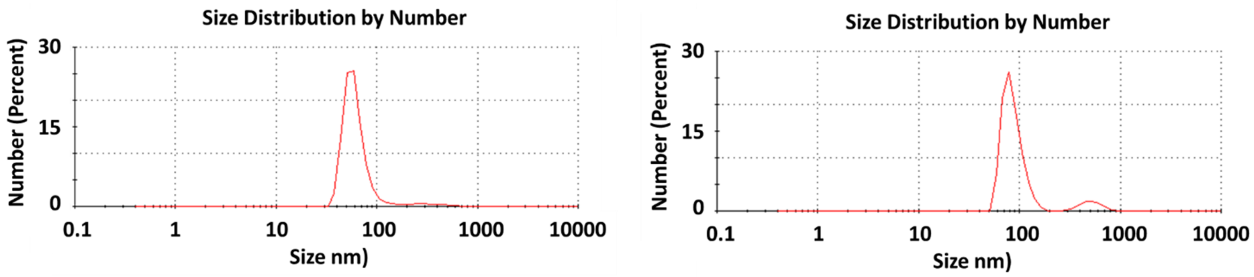

AuNP-SPTyr8 (0.25 mg/mL hTf)

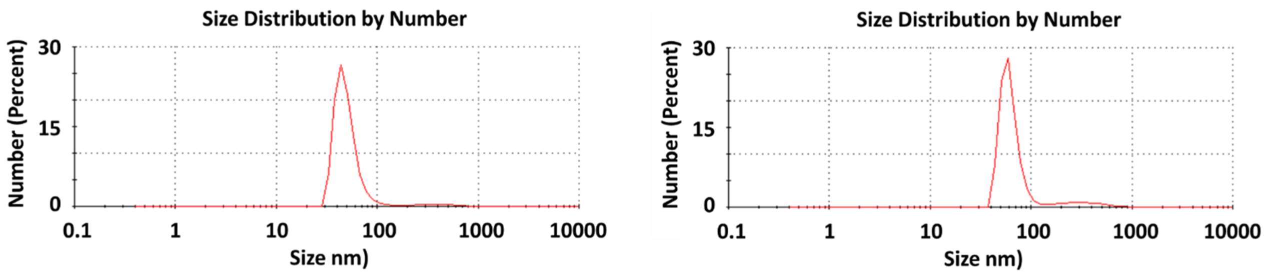

Figure S7. *Cont.* Size distribution by number of particles of AuNP-SPTyr8 in the presence of HSA and hTf proteins in the concentration range 0.015–0.25 mg/mL. DLS results are given for two independent measurements.

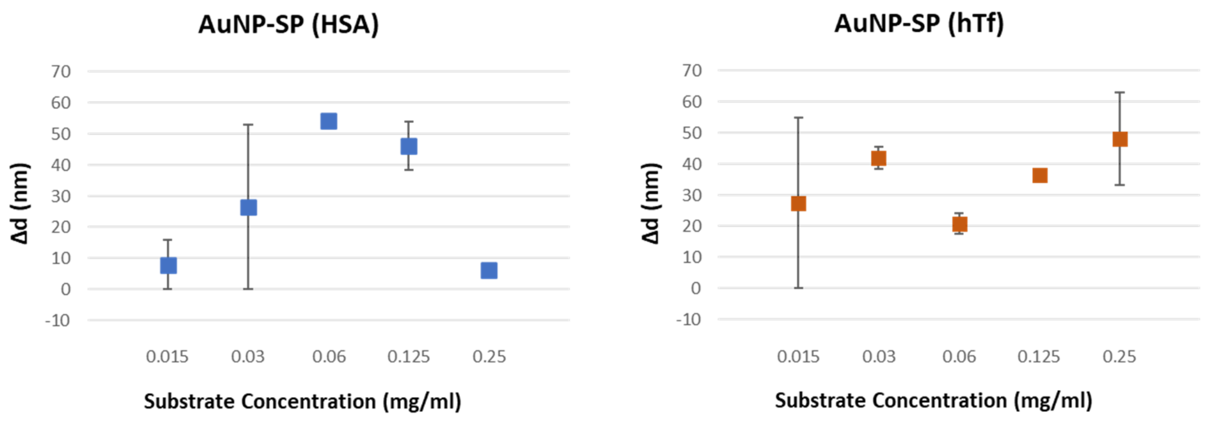

Figure S8. Variation of the hydrodynamic size for AuNP-SP nanoparticles in the presence of increasing concentrations of HSA (**left**) and hTf (**right**).

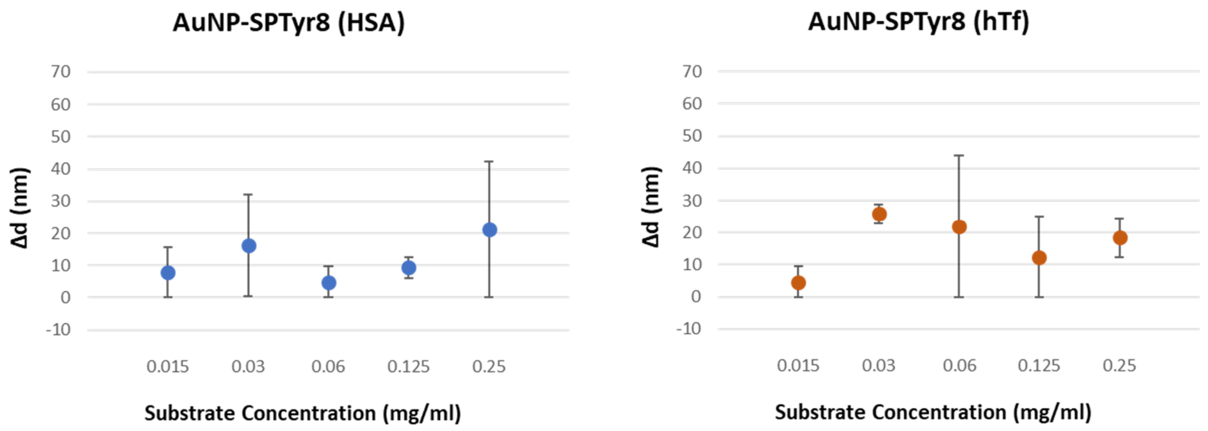

Figure S9. Variation of the hydrodynamic size for AuNP-SPTyr8 nanoparticles in the presence of increasing concentrations of HSA (**left**) and hTf (**right**).

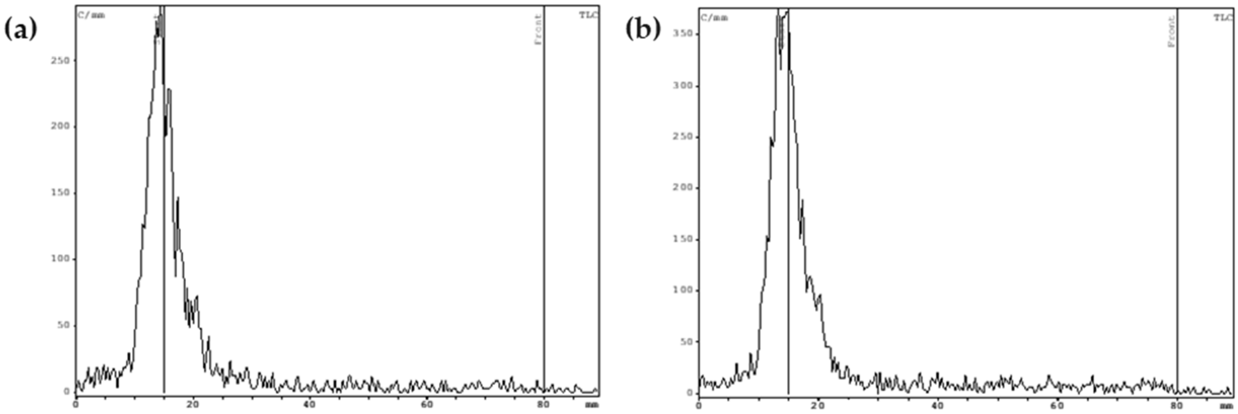

Figure S10. Radiochromatograms (ITLC-SG, 6M HCl /MeOH (5:95)) of ^{67}Ga-AuNP-SP (**a**) and ^{67}Ga-AuNPs-SPTyr8 (**b**) (^{67}Ga-AuNPs, R_f = 0.0; $^{67}Ga^{3+}$, R_f = 1.0).

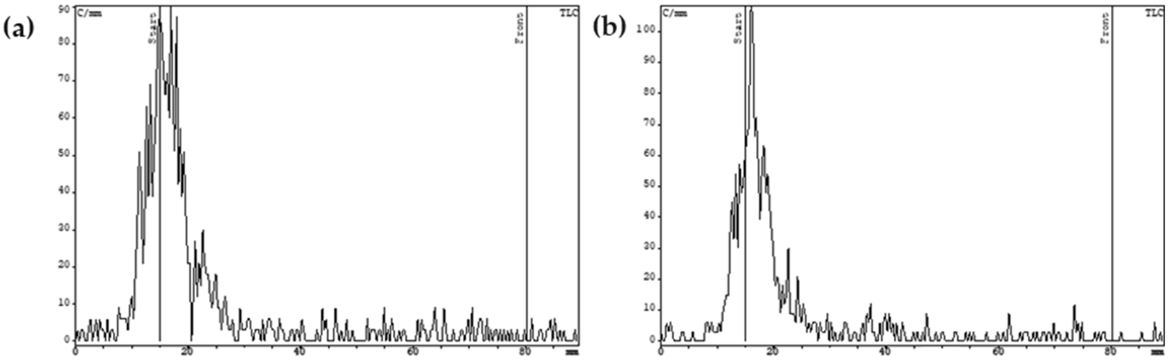

Figure S11. Radiochromatograms (ITLC-SG, 6M HCl /MeOH (5:95)) of ^{177}Lu-AuNP-TDOTA (**a**) and ^{177}Lu-AuNPs-SPTyr8 (**b**) (^{177}Lu-AuNPs, R_f = 0.0; $^{177}Lu^{3+}$, R_f = 1.0).

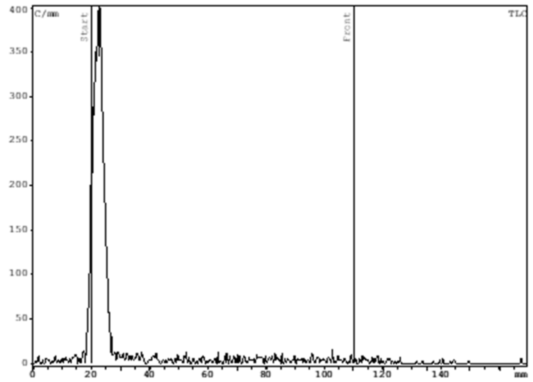

Figure S12. Radiochromatogram (ITLC-SG, 6M HCl /MeOH (5:95)) of ^{125}I-AuNPs-SPTyr8 after purification (^{125}I-AuNPs, R_f = 0.0; $^{125}I^-$, R_f = 1.0).

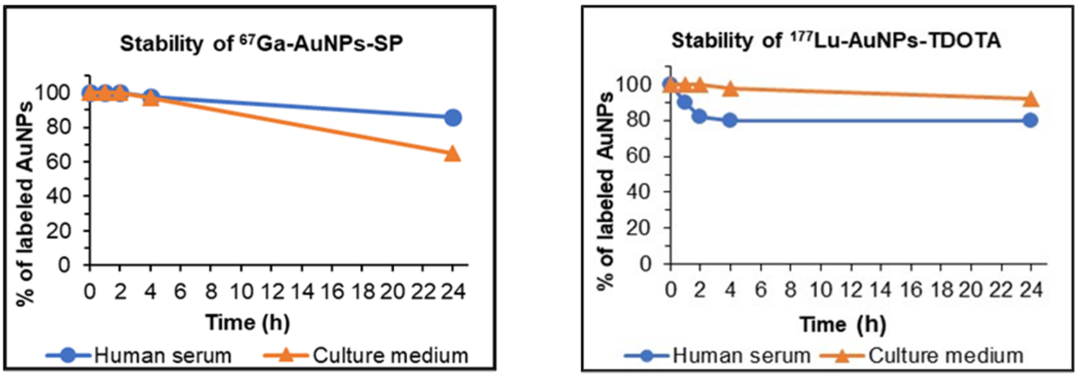

Figure S13. Radiochemical stability of ^{67}Ga-AuNP-SP (left) and ^{177}Lu-AuNP-TDOTA in the presence of human serum and cell culture medium, at 37 °C.

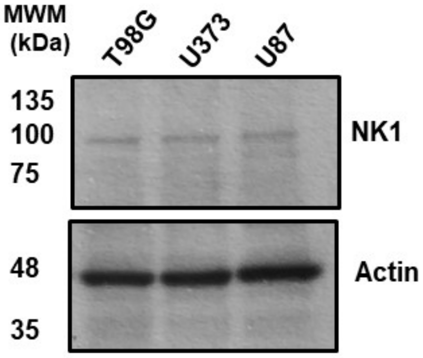

Figure S14. Western blot of NK1R in GBM cell lines. The ~100 kDa band detected corresponds to glycosylated NK1R. Actin was used as a loading control.

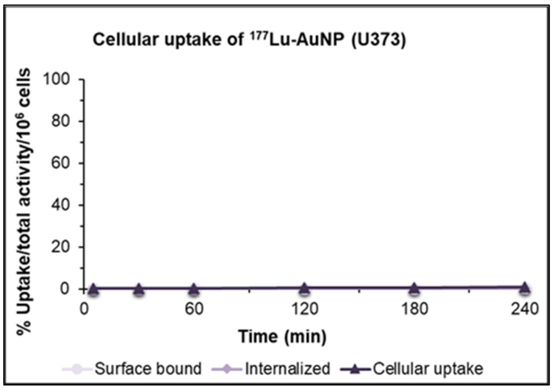

Figure S15. Cellular uptake and internalization of ^{177}Lu-AuNP-TDOTA in U373 cells, at 37 °C and different incubation times, determined by γ-counting measurements. Results are expressed as the percentage of the total (applied) radioactivity (mean ± SD) and normalized per million of cells.

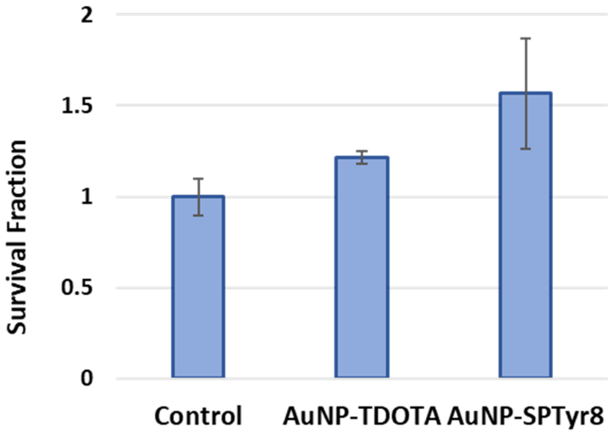

Figure S16. Results of clonogenic assays of U373 cells treated with AuNP-TDOTA or AuNP-SPTyr8, used at the maximum gold concentration applied in the clonogenic assays with the ^{177}Lu-labeled congeners, in comparison with untreated control cells.

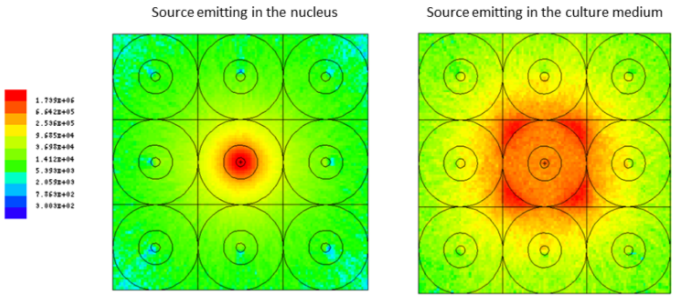

Figure S17. Example of IC electron flux distribution in the case of: (**left**) radionuclide emitting in the nucleus and (**right**) emitting in the culture medium around the central cell.

Table S1. Cell dose assessment when ^{177}Lu is emitting only in the culture medium.

Applied Activity (MBq)	Average Cell Dose–Scenario CMO (Gy)
0.03	0.014
0.9	0.043
1.8	0.086
